# Molecular determinants of ligand efficacy and potency in GPCR signaling

**DOI:** 10.1126/science.adh1859

**Published:** 2023-12-22

**Authors:** Franziska M. Heydenreich, Maria Marti-Solano, Manbir Sandhu, Brian K. Kobilka, Michel Bouvier, M. Madan Babu

**Affiliations:** 1 Department of Molecular and Cellular Physiology, Stanford University School of Medicine, Stanford, CA, USA; 2 MRC Laboratory of Molecular Biology, Cambridge, UK; 3 Department of Biochemistry and Molecular Medicine, Institute for Research in Immunology and Cancer, Université de Montréal, Montreal, QC, Canada; 4 Department of Pharmacology, University of Cambridge, Cambridge, UK; 5 Department of Structural Biology and Center of Excellence for Data-Driven Discovery, St. Jude Children’s Research Hospital, Memphis, TN, USA

## Abstract

G protein-coupled receptors bind to extracellular ligands and drugs and modulate intracellular responses through conformational changes. Despite their importance as drug targets, the molecular origins of pharmacological properties such as efficacy (maximum signaling response) and potency (the ligand concentration at half-maximal response) remain poorly understood for any ligand-receptor-signaling system. We used the prototypical adrenaline-β2-adrenergic receptor-G protein system to reveal how specific receptor residues decode and translate the information encoded in a ligand to mediate a signaling response. We present a data science framework to integrate pharmacological and structural data to uncover structural changes and allosteric networks relevant for ligand pharmacology. These methods can be tailored to study any ligand-receptor-signaling system, and the principles open possibilities for designing orthosteric and allosteric compounds with defined signaling properties.

## Introduction

Heterotrimeric guanine nucleotide-binding protein (G protein)-coupled receptors (GPCRs) constitute a major family of membrane proteins that respond to diverse extracellular ligands, including photons, small molecules, neurotransmitters, and hormones (1–5). In humans, over 500 endogenous ligands (6) act on 800 GPCRs and regulate key aspects of human biology (7, 8). As a result of their capacity to modulate human physiology, GPCRs are targets of about one-third of all FDA-approved drugs for various diseases (9). Ligand binding to a GPCR induces conformational changes that enable modulation of downstream signaling responses (10). During that process, the ligand shifts the conformational equilibrium of the receptor toward a more active or inactive state, depending on whether it is an agonist, antagonist, or inverse agonist (11, 12). The conformational states associated with agonist binding strongly increase the likelihood of a signaling response (e.g., G protein activation). This agonist capacity to promote signaling has been classically quantified by two fundamental pharmacological parameters: efficacy and potency (13), which can be determined by monitoring the receptor signaling response at various ligand concentrations (concentration-response curves). Efficacy relates to the maximum amplitude of a signaling response, whereas potency refers to the agonist concentration at which signaling reaches the half-maximal response. The tertiary structure of GPCRs has been subject to evolutionary selection pressure, resulting in endogenous agonist efficacy and potency to be within a physiologically relevant range. Thus, understanding how the ligand interacts with the receptor to modulate the signaling response is paramount to rationalizing the link between endogenous ligand properties and receptor sequence and structure, which can aid in designing drugs that engage specific receptor residues to elicit distinct signaling responses in a disease context.

Numerous structural studies have revealed the importance of the movement of specific transmembrane helices as a key feature that is associated with receptor activation upon ligand binding (14–16). Furthermore, changes in the size of the ligand binding cavity have been associated with high-affinity ligand binding and the strength of the signaling response (17). Extensive mutagenesis studies have identified residues along the receptor sequence that are important for modulating downstream signaling activity (18, 19). Despite the thorough pharmacological, structural, and mutational characterization of numerous ligand-GPCR pairs over the years, we do not yet fully understand how specific residues of the receptor decode and translate the information that is encoded in the ligand atoms to mediate a distinct intracellular signaling response (i.e., the *molecular origins* of efficacy and potency). For instance, in the well-studied adrenaline-β2-adrenergic receptor (β2AR)-Gs signaling system, it is unclear whether all residues that contact the ligand or the G protein (Fig. S1) are important for adrenaline’s efficacy and potency towards Gs and whether all residues that are involved in structural changes during activation are important for signaling (Fig. S1). If not, it would be helpful to identify the residues that help convert ligand binding into a signaling response and how they contribute to the efficacy and potency response of the ligand at the receptor.

**Fig. 1:**
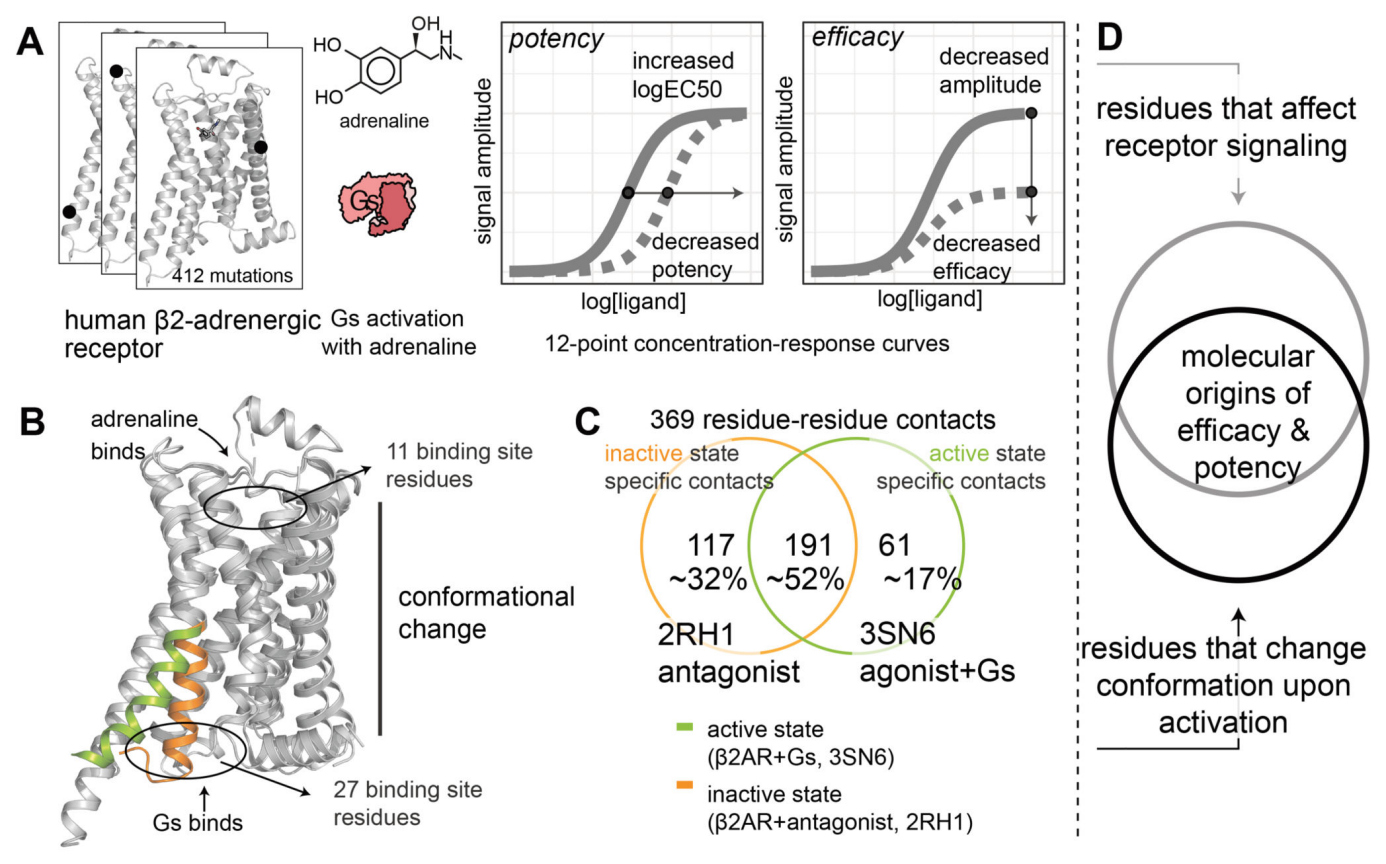
An approach to integrate pharmacological and structural data to reveal the molecular determinants of efficacy and potency. (**A**) Every residue of a receptor is mutated, and key pharmacological properties of the adrenaline-β2-adrenergic receptor-Gs signaling system are determined experimentally. (**B, C**) Active and inactive-state structures of receptors are analysed as networks of non-covalent contacts between residues to infer contacts that are specific for the active and/or inactive state. (**D**) These data are integrated using a data science approach to discover the molecular determinants and the underlying allosteric network governing efficacy and potency.

To systematically infer how ligand atoms are decoded by the receptor sequence and structure to modulate downstream signaling response, one needs to perturb the side chain of every receptor residue, reliably measure the impact on efficacy and potency for a signaling response, and contextualize the structural changes of the receptor residue with its pharmacological importance. We used β2AR, a well-studied, prototypical Family A GPCR that mediates "fight or flight" response when activated by its endogenous agonist adrenaline (epinephrine) and signals through the G protein Gs. By developing a data science framework that integrates (i) experimentally measured values of adrenaline-stimulated Gs signaling upon systematically perturbing the side-chain of every residue in β2AR and (ii) data on structural changes associated with agonist binding, receptor activation, and Gs coupling (Fig. 1), we reveal the molecular origins and principles governing ligand efficacy and potency in this prototypical GPCR signaling system.

## Results

### BRET-based biosensors allow in-depth pharmacological profiling of receptor mutants

We perturbed the sidechain of each of the 412 residues in the receptor by mutating the residue to alanine, or glycine if the native amino acid was alanine, as both types of substitutions are well tolerated in the long, membrane-spanning α-helices (20) of a GPCR. For each mutant, we then evaluated its adrenaline-stimulated Gs signaling profile in a live-cell assay with a BRET (Bioluminescence resonance energy transfer)-based biosensor (21, 22) (Fig. S2, see **Methods**). The biosensor reports on the distance between the Gα and Gγ subunits of the heterotrimeric G protein; this distance increases upon receptor activation due to conformational changes in the α subunit and its dissociation from the βγ-subunits, leading to a decrease of resonance energy transfer between the donor and acceptor that are fused to the α and γ subunit, respectively. Each assay was performed as 12-point concentration-response curves in biological triplicates (Fig. 1, top panel, **Methods**) to quantify the signal amplitude (efficacy) and logEC50 (potency) of the adrenaline-stimulated G protein activation (Fig. S2 and **Methods**). Because a mutation may affect receptor abundance and low cell-surface abundance could negatively influence the signaling, we also evaluated the abundance of all mutants by cell-surface enzyme-linked immunosorbent assay (Fig. S3A-C, see **Methods**). We performed measurements on cells expressing wild type β2AR or no receptor as controls. In total, we obtained over 16,000 data points; the pharmacological properties for agonist-induced Gs activation for each mutant are reported in Table S1. For subsequent analyses of these data, we used the GPCRdb numbering scheme (23), a system based on Ballesteros-Weinstein numbering (24) wherein the first number in superscript refers to the helix or loop, and the second number refers to the position relative to the most conserved residue (numbered 50) in that helix (e.g. L124^3x43^, which is 7 residues before the most conserved residue in TM3).

### One-fifth of the positions in β2AR are important for adrenaline efficacy, potency, or both

**Fig. 2:**
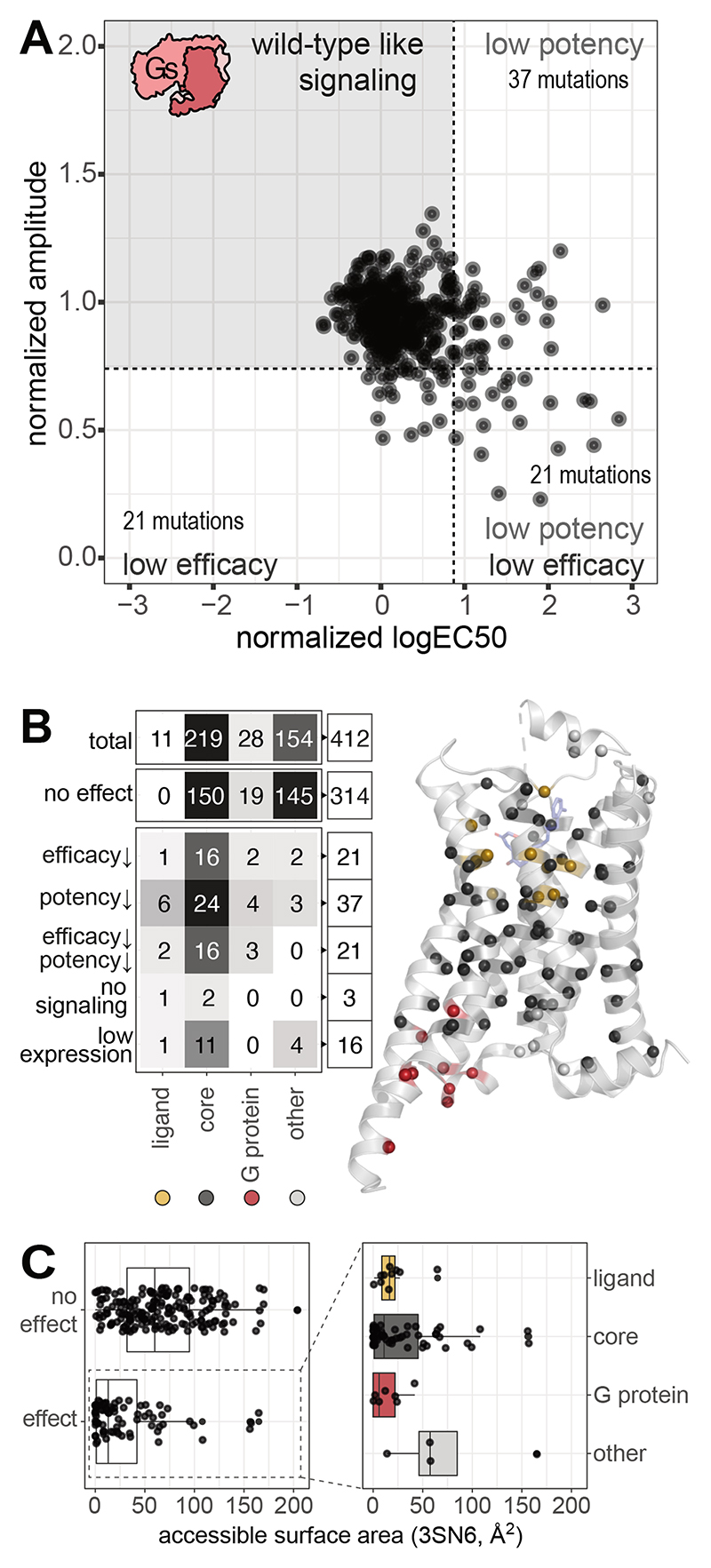
Receptor positions that affect efficacy or potency upon mutation. (**A**) Overview of potency (normalized logEC50) and efficacy (normalized signal amplitude) of all β2AR mutations. Cut offs are shown as dashed lines (**Methods**). (**B**) Distribution of residues by functional relevance and their effect on efficacy and potency (**Methods**). Ligand binding pocket and G protein–binding site include residues within 4 Å of adrenaline (PBD 4LDO) or Gs (PDB 3SN6), respectively. (**C**) Accessible surface area of residues, grouped by whether a mutation affects signaling (‘effect’) or not (‘no effect’). For residues where signaling was affected by mutation, the location of mutations in the ligand binding site, core, G protein binding site or in none of the above (‘other’) is indicated.

For 16 receptor positions, sidechain perturbation severely affected cell surface abundance (<25% of WT level; **Methods**). These mutants were excluded from further analysis as we could not reliably determine whether the observed reduction in signaling resulted from altered function, reduced abundance, or both (Fig. S3A-C). Many of these positions are highly conserved and are likely important for protein folding, receptor biogenesis, and transport to the cell membrane. (Fig. S3D-H). The remaining 396 mutants had cell-surface abundance that allowed reliable estimation of pharmacological parameters and were therefore considered for further analysis (abundance >25% of the wild-type β2AR; **Methods**). Most of the single-point mutations (~80%; at 314 positions) did not negatively affect Gs signaling, and ~20% (at 82 positions) had an impact on signaling. Of these 82, 21 primarily reduced efficacy, 37 primarily reduced potency, 21 reduced both potency and efficacy, and 3 resulted in no measurable signal (Fig. 2A-B).

Mapping these positions on the secondary structure elements of the receptor showed that TM5 was enriched in mutations that affected potency only, whereas TM3 was enriched in mutations that affected efficacy only. The mutations that affected efficacy and potency simultaneously were distributed across the different transmembrane helices (Fig. S4A-D). Although some of the identified positions overlap with functional residues in the ligand-binding pocket and G protein-binding interface, several positions mapped to other parts of the receptor, including residues on the receptor surface, with the highest density mapping to the receptor core (Fig. 2B-C). These findings collectively indicate that the efficacy and potency of this endogenous agonist at this receptor are likely governed by a subset of receptor positions (~20%) that are not just restricted to known functional sites but also distributed in different parts of the structure. These receptor positions thus decode and translate ligand binding into a signaling response and are, hence, pharmacological determinants for this ligand-receptor-signaling system.

### Each receptor residue-ligand atom contact contributes differentially to efficacy and potency

**Fig. 3:**
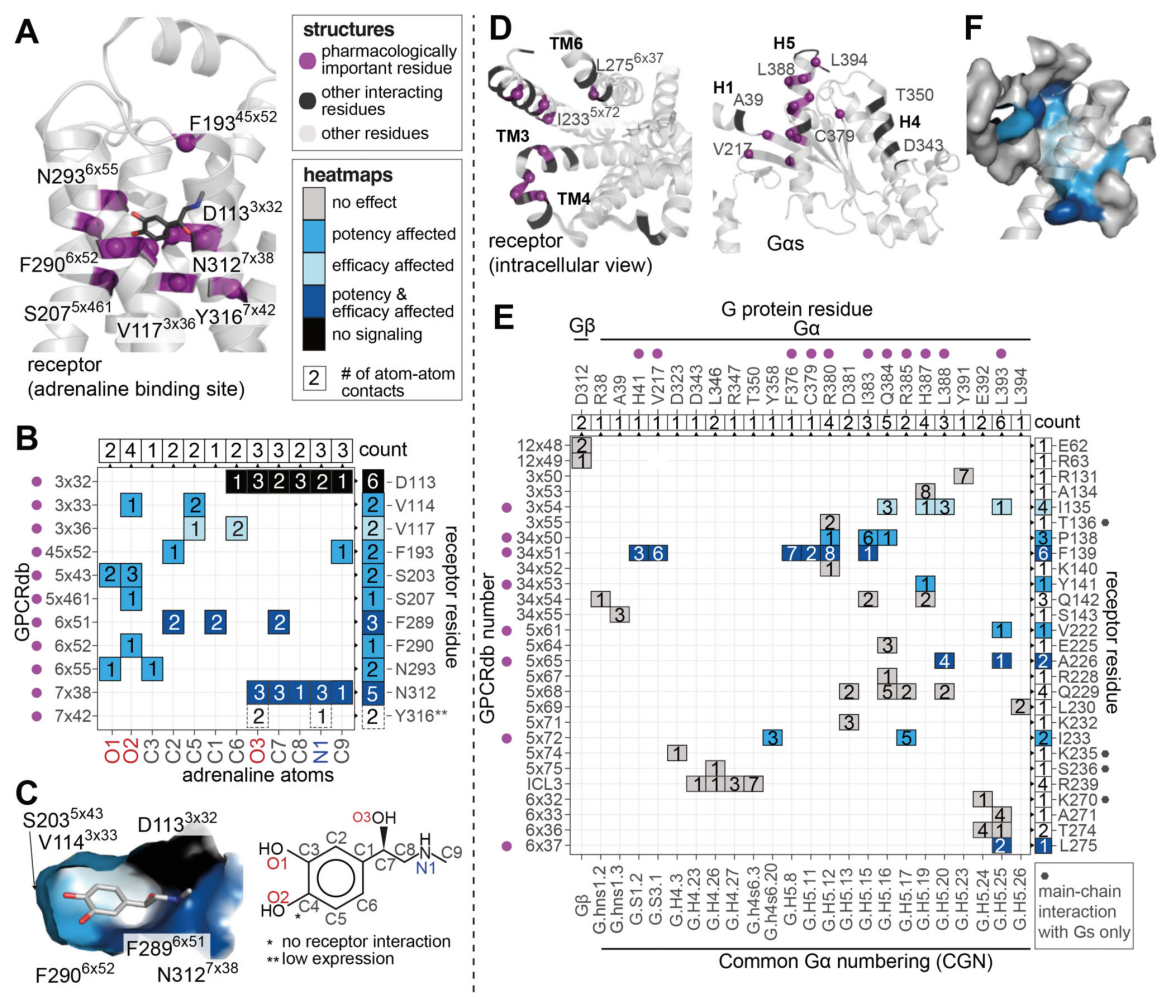
Effect of mutations in the ligand- and G protein-binding sites. (**A**) View of the ligand-binding site, positions are labeled with residue number and GPCRdb number in superscript. Pharmacologically important residues (according to cut-off values, see **Methods**) are indicated in violet. Not labeled for clarity: V114^3x33^, S203^5x43^, F289^6x51^. (**B**) Receptor residue-ligand atom contact plot with the ligand atoms on the x-axis and the receptor’s GPCRdb and residue numbers on the y-axis. The number of non-covalent contacts between a receptor residue and ligand atom is shown in each square of the heatmap. The chemical structure of adrenaline below the heatmap indicates the labeling of adrenaline atoms used for the x-axis. Box colors in the heatmap refer to the pharmacological effect of the mutation (efficacy affected (light blue), potency affected (ocean blue), both efficacy and potency affected (dark blue), no measurable signaling (black)). The number of receptor residues contacted by each ligand atom and ligand atoms contacted by each receptor residue are indicated in boxes at the top and right-hand side of the heatmap, respectively. (**C**) Surface view of the ligand-binding site, where the surface of each receptor residue is colored by the effect its mutation had on signaling. (**D**) Views of the G protein-binding site on the receptor and the Gs protein. Positions are labeled with residue number and, with GPCRdb numbers. Pharmacologically important residues are indicated in violet and other interacting residues are marked in black. (**E**) Receptor-G protein residue contact plot based on the structure of β2AR in complex with heterotrimeric Gs (PDB 3SN6). G protein residues are shown on the x-axis and the receptor residues on the y-axis. The receptor residues that contact the G protein through a receptor main-chain contact only are marked with a dark grey hexagon on the right side of the matrix. The number of non-covalent contacts between a receptor residue and a G protein residue are mentioned in each square of the heatmap. See legend for panel **B** for box colour description. The CGN number (Common G protein numbering scheme) is provided on the x-axis. The number of contacting residues by the receptor and the G protein are shown on the top and right-hand side of the heat map. Residue T68 is not included as it is a contact that is observed only in the structure of β2AR with Gs peptide (PDB 6E67). (**F**) Surface view of the G protein binding site coloured by mutational effect with helix 5 shown as cartoon.

We investigated how each ligand-contacting residue contributes to efficacy and potency. For this, we first identified all receptor residues that contact adrenaline (PDB 4LDO, (25)) (Fig. 3A; **Methods**) and the ligand atoms they contact by constructing a receptor residue-ligand atom contact matrix (Fig. 3B). Although mutants of all 10 of the 11 adrenaline-contacting residues that expressed well negatively affected the signaling response, they had distinct effects on efficacy and potency (Y316A^7x42^ drastically decreased receptor abundance; Fig. S3). Mutations in around half of the positions (6 of 10) affected potency only, one affected efficacy only, two affected both efficacy and potency when mutated, and D113^3x32^A completely abolished signaling. D113^3x32^ is critical for agonist binding in β2AR (26, 27) and other aminergic receptors, as it contacts the positively charged amine group, which is a common feature of endogenous agonists for monoamine receptors. When this information was mapped onto the ligand-binding pocket (Fig. 3C), it was obvious that the ligand-contacting residues that affect potency upon mutation only group together on one side of the receptor involving TMs 3, 5, and 6, whereas those that affect both efficacy and potency upon mutation group together on the other side of the receptor, largely involving TMs 6 and 7. The ligand-contacting residue that affects efficacy only is in TM3 and deeply buried in the binding pocket. These results indicate that each receptor residue that contacts the ligand contributes differentially to the overall pharmacological response. Of the 159 Å^2^ of receptor surface area occupied by the ligand, 20% (31.5 Å^2^) appears to be important for both efficacy and potency, 6% (9 Å^2^) for efficacy only, and 59% (93 Å^2^) for potency only. Thus, the pharmacological parameters of a ligand at a receptor seem to emerge from the differential contribution of each receptor residue to the overall efficacy and potency in response to a ligand.

From the ligand perspective, some adrenaline atoms (the two hydroxyl groups attached to the benzene ring and C3; Fig. 3B) uniquely mediate non-covalent contacts with residues that only affect potency when mutated. A few ligand atoms contact residues that either affect potency only, or efficacy only or both when mutated (C5, C2, C6, and C1 of the benzene ring). The remaining ligand atoms make extensive contact with two residues that affect both efficacy and potency when mutated, including the N312^7x38^ and the key D113^3x32^ (O3, C7, C8, N1, and C9). These findings reveal the existence of spatially clustered groups of adrenaline atoms that mediate contact with specific receptor residues to preferentially affect efficacy and/or potency responses in receptor signaling. An implication of this observation is that chemical modifications of these atoms that can mediate contacts with specific receptor residues might allow a more targeted ligand modification to alter the efficacy or potency of Gs activation. This can be seen from the receptor residue-ligand atom contact matrix for the β2-adrenergic ligand salmeterol (Fig. S5). Together, these results indicate that, although all ligand contacting residues are important for receptor signaling, distinct receptor residues and specific chemical groups on the ligand contribute differentially to efficacy and potency responses. Thus, although efficacy and potency are measured as a general ligand property at a receptor, they arise from a collection of receptor residue-ligand atom non-covalent contacts, each of which contributes differentially to pharmacological properties in downstream signaling response.

### Only one-third of the G protein-contacting residues are important for efficacy and potency

From the structures of the β2AR-Gs complex (PDB 3SN6 and 6E67), we observed that 27 receptor residues make 49 non-covalent contacts with 24 G protein residues (Fig. 3D-F, **Methods**). Only a third (9 of 27) of the receptor interface residues showed an effect on G protein activation when mutated. This is in stark contrast to the ligand binding residues, where all positions were important for receptor signaling (Fig. 3B). Of the positions in the G protein binding site, four affected potency only, two affected efficacy only, and three affected both when mutated. From the G protein perspective, key conserved residues that undergo conformational changes during receptor binding (e.g., H5.12,15,16,19,20,25, S3.1, and S1.2 in the CGN numbering scheme (28)) mediate extensive contacts with receptor residues that affected efficacy, potency, or both when mutated (28, 29). Collectively, these results reveal that there is a subset of receptor-interface residues that translate ligand potency and efficacy into G protein activation at the receptor-G protein interface. A large fraction of receptor residues that contact the G protein can be mutated to alanine with little effect on efficacy or potency and are thus evolvable. The latter findings imply that upon gene duplication and divergence, receptors can tolerate certain types of mutations at the G protein–binding interface without losing their capacity for signal transduction. Such a structure-to-function architecture may provide an additional explanation as to why GPCRs could evolve by gene duplication and acquire new G protein selectivity (30, 31).

### Conserved motifs only explain a fraction of residues important for signaling

Of all the positions that had an influence on ligand efficacy and potency, only a fraction (19 of 82 residues, 24%) were in the ligand-binding and G protein-binding sites. A detailed analysis of the key motif residues of GPCRs, as well as the most highly conserved positions (x50 positions in each helix) (**Methods**), accounted for an additional 8 residues (10%)(Fig. S6). Key sequence and structural motifs with known functional roles include CWxP, PIF, NPxxY, and DRY (where each uppercase letter is an amino acid residue and each ‘x’ represents any amino acid). However, two-thirds of the pharmacologically important residues (55 of 82 residues) do not map to known functional sites or motifs and are hence not readily interpretable. These positions most likely represent receptor- or receptor family-specific switches or allosteric conduits that contribute to translating ligand binding into signaling.

### Structural changes alone do not specify the pharmacological importance of a residue

**Fig. 4:**
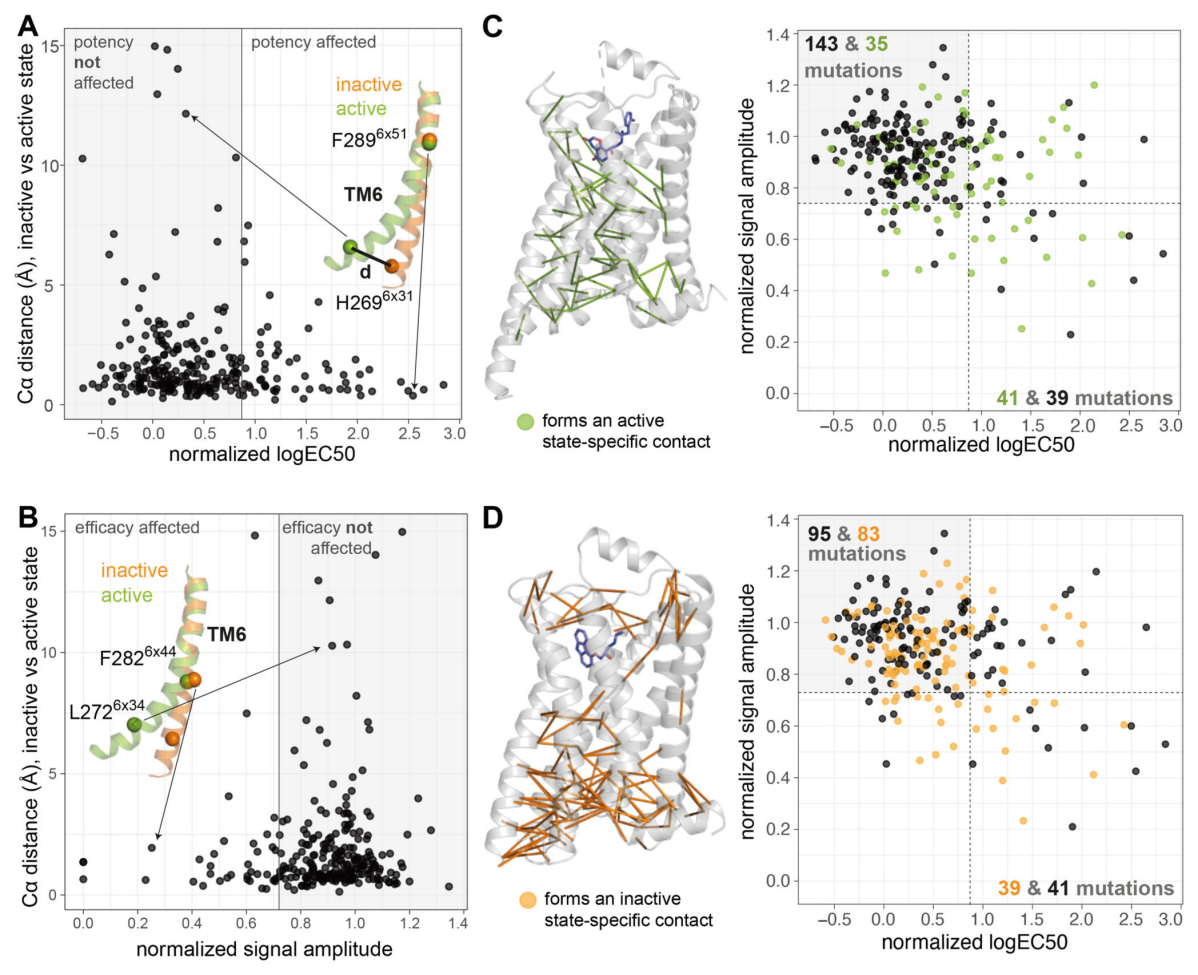
Structural changes and pharmacological importance of residues. (**A**) Distance between Cα atoms in the inactive state (PDB ID 2RH1) versus the active, G protein-bound state (PDB ID 3SN6) plotted against normalized Gs logEC50 of the alanine mutant in that position. White and grey areas indicate residues with potency affected and not affected, respectively, upon mutation. (**B**) Distance between Cα atoms plotted against normalized Gs signal amplitude. White and grey areas indicate residues with efficacy affected and not affected, respectively, upon mutation. (**C**) Active state-specific contacts (see **Methods**) are shown on the active, G protein-bound receptor structure. Potency and efficacy values for all mutants are plotted (except those with low abundance and those for which no signaling was detected). Mutants are colored green if the mutated residue is involved in an active state-specific contact and black if it is not. White areas indicate residues with efficacy, potency, or both affected, and the grey area indicates those that were not affected upon mutation. Numbers in the plots denote the number of residues in each category (black or green). (**D**) Inactive-state specific contacts on the inactive-state structure. A mutant (data point in the plot) is colored orange if the residue is involved in an inactive state-specific contact and black if it is not. White and grey areas of the graph are defined as in **C**.

Because key structural changes have been extensively described as a hallmark of receptor activation (32–35), we investigated how those structural changes are associated with pharmacologically important residues. We calculated how much each residue moved during activation by computing the distance between the α-carbon atoms of each residue in the inactive (highest resolution structure, 2.4 Å, PDB 2RH1) and active, G protein-bound state (highest resolution structure, at 3.2 Å, PDB 3SN6). The distribution of the distance moved by each residue is skewed to the right, where in ~8% of the cases, the α-carbon atom moved more than 4 Å (22 residues, Fig. S1B). Of these, only 7 were pharmacologically important and affected efficacy or potency when mutated, whereas the rest had no impact on downstream signaling (Fig. 4A-B). Furthermore, comparing the set of residues that were pharmacologically important from those that were not revealed that an approximately equal proportion between the two sets appeared to undergo a structural change (i.e. move > 4 Å). Finally, an analysis of dihedral angles in the active and inactive states also revealed no association between the extent of change in the dihedral angle and pharmacological importance of a residue (Fig. S7). These findings collectively suggest that it is not possible to infer the pharmacological importance of a residue just from the extent of structural change (translation or rotation) during receptor activation.

Because the formation or breaking of non-covalent contacts during activation comprises both translational (distance) and rotational (dihedral angle) changes to different extents, we focused on residues that make active state–specific contacts or break inactive state–specific contacts to assess whether they can inform pharmacological importance (Fig. 4C-D, **Methods**, Table S2). Considering those two categories, the formation of an active state–specific contact by a residue showed a higher association with pharmacological importance. For instance, about 50% (41/80 residues with structural data) of residues that are pharmacologically important participated in an active state–specific contact, whereas only 20% (35/178) of the pharmacologically unimportant residues did. This effect was not observed for inactive state–specific contacts, for which ~50% (39/80) of pharmacologically important residues with structural data and 47% (83/178) of unimportant residues participated in an inactive state–specific contact. These findings indicate that systematic integration of mutational effects and structural analyses considering active state–specific residue contacts may help identify and characterize the subset of pharmacologically important residues that undergo a structural change in this ligand-receptor system. Importantly, it can explain which of the structural changes that happen during activation are relevant for ligand efficacy and potency at the receptor.

### Linking structural changes with pharmacological importance reveals new roles for residues

**Fig. 5:**
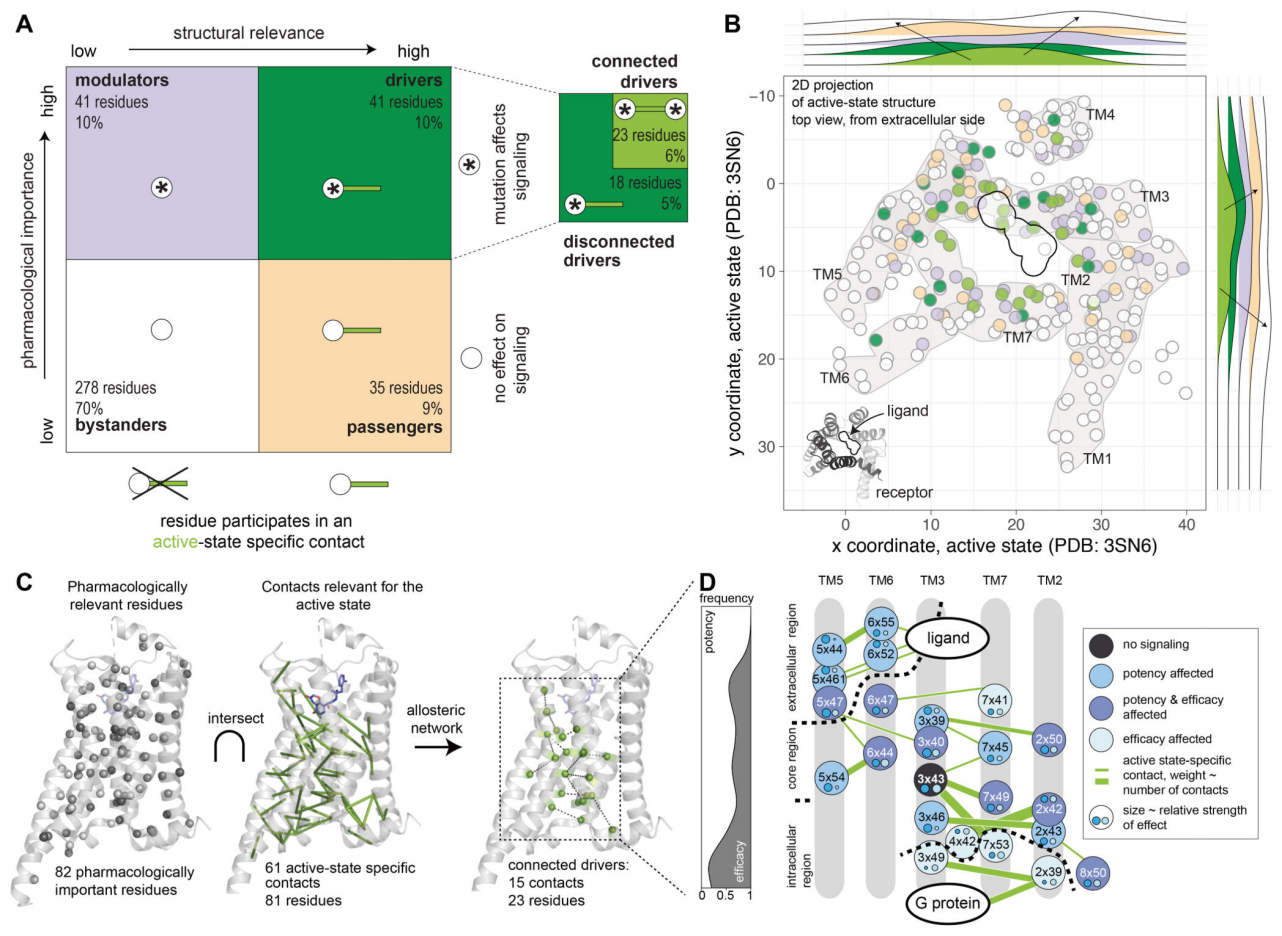
The allosteric contact network for efficacy and potency as determined by structure and pharmacology. (**A**) Classification of residues according to their pharmacological importance (whether a mutation affected signaling or not) and structural relevance (whether a residue forms an active-state specific contact or not) into four main classes: bystanders (white), passengers (wheat), modulators (slate) and drivers (green), the latter is subdivided into connected drivers (driver residues connected to other driver residues) and unconnected driver residues (**Methods**). (**B**) Position of residues classified by their participation in active state-specific contacts and importance for pharmacology on a 2D top view projection of the active, G protein-bound structure of the β2AR (PDB 3SN6). Frequency of residues along the x- and y-axis of the receptor (plane parallel to the membrane) is shown on the distributions outside. Arrows denote the direction of the peaks of the distribution for the residue classes. Driver residues tend to be in the centre, whereas bystander residues tend to be at the periphery. (**C**) Integration of pharmacologically important residues and the residues contributing to newly established contacts in the active-state structure yields the allosteric network for efficacy and potency (see **Main text**). (**D**) Residues forming the allosteric networks for efficacy and potency are represented as a cartoon, colored by the effect of the mutation. The graph (left panel) depicts the frequency of potency versus efficacy effects for residues in the network, projected on the axis running along the extra- to intra-cellular side. Small circles and their size (scaled to the relative strength of effect) within each residue indicate the magnitude and effect on potency and efficacy. Edge thickness in the network corresponds to the number of contacts, ranging from 1-5.

To identify the structural changes relevant for efficacy and potency, we devised an approach to integrate the experimentally determined pharmacological parameters with the active state–specific contacts (Fig. 5A; **Methods**). We first classified every residue as *pharmacologically important* (if it affects efficacy, potency, or both when mutated) or *not pharmacologically important* (no effect on either efficacy or potency). We then stratified according to whether a residue forms an active state–specific contact or not (i.e. *structurally important* or *not*). Based on these two properties, we defined four classes of residues: (i) “driver residues” that mediate an active state–specific contact and affect pharmacology (41 residues), (ii) “modulator residues” that don’t form an active state–specific contact but are important for pharmacology (41 residues); (iii) “passenger residues” that mediate an active state–specific contact but are not important for pharmacology (35 residues) and (iv) “bystander residues” that neither mediate active state–specific contacts nor affect pharmacology when mutated (278 residues; **Methods**, Fig. 5A). Thus the pharmacologically important residues are either drivers or modulators (82 mutations that affect pharmacology), and the structurally important residues are either drivers or passengers (76 residues that mediate an active state-specific contact). As a general pattern, driver residues tend to be close to the central axis going through the receptor and perpendicular to the membrane, whereas modulator and passenger residues are located further away from this axis, in proximity with solvent or membrane-exposed positions (Fig. 5B). These observations prompted us to investigate the existence of a network of pharmacologically relevant active state-specific residue contacts mediated by driver residues, and further understand the role of modulator residues for signal transduction.

### A subset of residues driving efficacy and potency form an allosteric contact network

Of the 41 driver residues, 23 form 15 active state-specific non-covalent contacts to other driver residues with a shared pharmacological effect (**Methods**; connected drivers). This allosteric network of active state-specific contacts mediated by driver residues allowed us to describe how potency and efficacy signals are transmitted across the receptor structure at the residue level through coordinated structural rearrangements (Fig. 5C and Fig. S8). The network involving connected drivers started extracellularly in TM5 and TM6 and formed several contacts between TM5 and TM6, including with F282^6x44^ of the PIF motif (36, 37)(Fig. 5D) before reaching TM3 in the receptor core. TM3 further connected to TM7 and the most conserved residue in TM2, D79^2x50^ (26), which is part of the allosteric sodium binding motif (Fig. 5D)(38). Finally, the network reached residues in the intracellular region, including D130^3x49^ and Y326^7x53^ of the conserved DRY and NPxxY motifs in TM3 and TM7, respectively (Fig. 5D). Potency-affecting driver residues in the network were enriched at the extracellular side of the receptor involving TM5 and TM6 (total of 9 residues; Fig. 5D), and efficacy-affecting driver residues were enriched at the intracellular side of the receptor in TM7, TM2, and TM3 (total of 5 residues; Fig. 5D). Driver residues in the allosteric network that affected both efficacy and potency were enriched in the center of the receptor, connecting to potency-only and efficacy-only residues (9 residues; Fig. 5D). This raises the question as to how ligand binding to the receptor initiates and transmits information through the allosteric network to modulate downstream signaling.

### Key contact rewiring events initiate and transmit information to mediate signaling

To understand how information is transmitted in the adrenaline-β2AR-Gs signaling system, we analyzed the relation between the ligand- and G protein-binding residues and the allosteric network. Focusing on the ligand-receptor interface, the allosteric contact network included three residues directly located in the ligand-binding site (N293^6x55^, F290^6x52^, and S207^5x461^; all affected potency only when mutated); the other eight ligand-binding residues do not form active state–specific contacts. This highlights the existence of residues in the ligand binding pocket that directly participate in the allosteric network. It also highlights the distinct roles mediated by the ligand-contacting residues on the receptor: although several residues stabilize ligand-receptor interactions, a subset of three driver residues that also contact the ligand likely initiate the allosteric changes during receptor activation (Fig. 5, Fig. S9). The latter set of three residues makes more extensive contact with other receptor residues than with adrenaline. In contrast, ligand-binding residues that do not mediate active state-specific contacts (e.g., modulator residues D113^3x32^ and N312^7x38^) make substantial contacts with adrenaline and fewer contacts with other receptor residues (Fig. S9). Thus, some receptor and ligand atom contacts might contribute to the affinity of ligand binding, whereas others might have a more direct role in triggering the allosteric network to transmit the signaling response, perhaps via initiating a structural change and stabilizing a particular receptor conformation.

Focusing on the G protein-binding residues, of the nine that affected efficacy or potency when mutated (**Methods**), one residue (driver residue T68^2x39^) is part of the allosteric network. Although T68 does not mediate a contact in the receptor-heterotrimeric G protein complex structure, it does form a contact with Gs in the receptor-Gs peptide complex structure (39), which may represent an intermediate state in complex formation. Furthermore, L275^6x37^ (a disconnected driver that affects efficacy when mutated) mediates contact with a conserved residue on the G protein (H5.25; CGN Numbering scheme (28)). L275^6x37^ is held in place through inactive state-specific contact with I127^3x46^. Upon receptor activation, this inactive state contact is broken, allowing 6x37 to contact the G protein and for 3x46 to mediate an active state-specific contact with Y326^7x53^ (40). Interestingly, this non-covalent contact is between a residue that affects potency only (3x46) and one that affects efficacy only (7x53). Thus, the G protein-binding residues that affect efficacy and potency when mutated can be classified into those directly affecting the interaction with the G protein when mutated and those that are part of or connected to the allosteric network (Fig. S10). Together, these observations highlight the importance of key rewiring events in the allosteric network involving the switch from the inactive state–specific contacts to active state–specific contacts upon ligand binding and G-protein engagement for signal transduction (Fig. 5D and Fig. S10). It also highlights that as functionally relevant intermediate state conformations are structurally characterized, the roles of some of the residues identified can be interpreted in more mechanistic detail.

### Modulator residues near the allosteric network and functional sites modulate pharmacology

Of the 82 residues that were important for efficacy or potency, the role of two-thirds (54 of 82 residues; 41 are driver and 13 are modulators) could be explained as either involved in ligand or G protein binding, conserved motif residues, or driver residues forming the allosteric network or that mediate active state–specific contacts (Fig. S11). We then investigated the role of the remaining one-third of the residues that are pharmacologically important (28 of 82 residues) and categorized them according to their likely function (Fig. S11; these are the remaining 28 of the 41 modulator residues). Of those, the function of ten residues may be explained by their ability to directly contact and perhaps stabilize a residue in the ligand binding pocket or by being in the putative ligand entry pathway. Another six residues mediate non-covalent contacts with a driver residue (network modifier), and the function of seven modulators may be explained by lipid interaction. The roles for the remaining five residues were less clear (Table S1, column ‘explanation’). We analyzed conformations of the active and inactive states, but a deeper understanding of the role of modulator residues can be obtained through the investigation of intermediate conformational states, by considering the role of structured water molecules in stabilizing or mediating conformational transitions, or through the analyses of data describing receptor dynamics (41, 42). Taken together, these results identify and describe how residues around the allosteric network and functional sites may modulate ligand efficacy and potency in β2AR.

### Surface-exposed driver, modulator, and passenger residues represent key allosteric sites

**Fig. 6:**
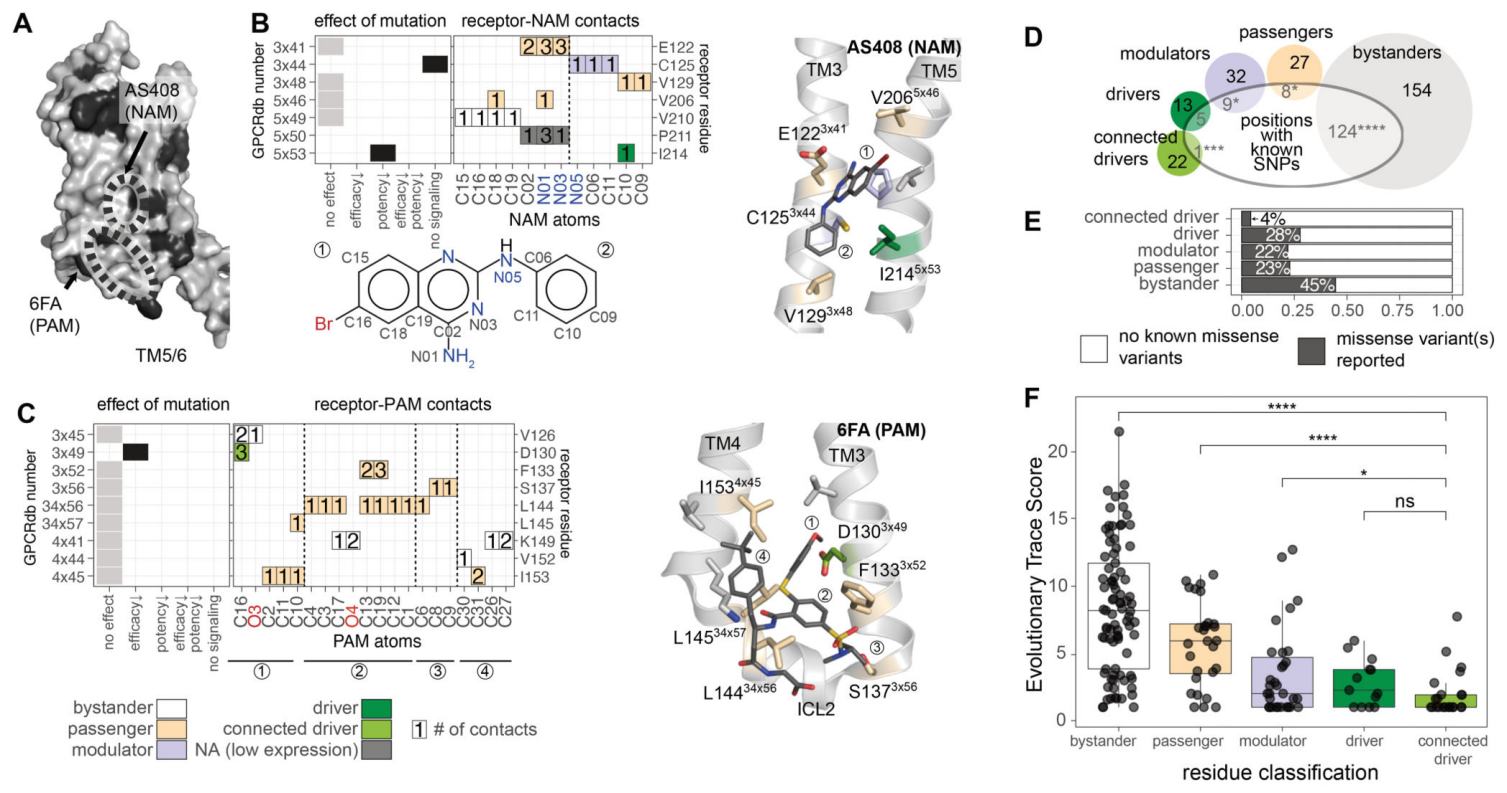
Allosteric sites, evolutionary conservation, and natural variation. (**A**) Surface-exposed pharmacologically important residues are shown in black on the β2AR (PDB ID: 3SN6). (**B**) Effect of mutations in the binding site for AS408, a negative allosteric modulator, receptor residue – ligand (NAM) atom contact plot, and structural view. The contact plot shows the number of contacts between receptor residues (y-axis) and NAM atoms (x-axis). Colors for the receptor-NAM contacts in the plot refer to the classification of the residues as in Fig. 5. The chemical formula of the allosteric modulator below the heatmap indicates the labeling of NAM atoms used for the x-axis of the contact plot. (**C**) Effect of mutations in the binding site for 6FA, a positive allosteric modulator, receptor residue – ligand (PAM) atom contact plot, and structural view. The contact plot shows the number of contacts between receptor residues (y-axis) and PAM atoms (x-axis). Colors for the receptor-PAM contacts in the plot refer to the classification of the residues as in Fig. 5. The circled numbers below the x-axis refer to different parts of the modulator and are mapped on the structure view for reference. (**D**) The number of residues with known single nucleotide polymorphisms (SNPs) for each class. (**E**) Percentage of residues for which at least one SNP has been reported by class. Significant differences from the expected frequency by chance are indicated according to a hypergeometric test (* p ≤ 0.05, ** p ≤ 0.01, *** p ≤ 0.001, **** p ≤ 0.0001, ns: non-significant). (**F**) Adrenergic receptor evolutionary trace (ET) score for the different residue classes. Groups were compared using a Wilcoxon test (* p ≤ 0.05, ** p ≤ 0.01, *** p ≤ 0.001, **** p ≤ 0.0001, ns: non-significant). Driver refers to the subset that is in the disconnected driver group.

Some of the pharmacologically important positions mapped to surface-exposed sites that were not part of the orthosteric ligand-binding site or the G protein-binding interface (Fig. 2C). The identification of pharmacologically important residues that lie on the receptor surface suggested possible structural sites for modulating receptor signaling that could be targeted by natural allosteric molecules (43, 44). To test whether lipids such as cholesterol exert effects allosterically by binding to such sites and altering signaling, we analyzed structures of receptors in complex with cholesterol (**Methods** and Fig. S12). Cholesterol bound to a site containing three modulator and three passenger residues on the receptor surface. Thus, surface-exposed pharmacologically important residues might be targeted by synthetic allosteric ligands. In line with this idea, an analysis of the structure of the receptor in complex with the negative allosteric modulator (NAM) AS408 revealed that its binding interface contained two amino acids, I214^5x53^ (driver) and C125^3x44^ (modulator) that are important for receptor signaling and three passenger residues, all of which are surface exposed (Fig. 6A-B; **Methods**; 7 residues contact the NAM). Similarly, an analysis of the structure of the receptor in complex with the positive allosteric modulator (PAM) 6FA showed that it bound to a different exposed surface containing the driver residue, D130^3x49^, which is important for receptor signaling (Fig. 6A-C). Five of the nine residues in the PAM binding site were passengers, i.e., residues that mediate active state–specific contacts but that do not affect efficacy or potency when mutated (Fig. 6A-C). Passenger residues were significantly overrepresented (compared to random expectation) in both PAM and NAM binding sites, even when the N- and C-termini were excluded from the test (p-value: 1.5 x 10^-3^, hypergeometric test).

These results show that allosteric modulators, which were discovered through screening efforts, tend to bind surface exposed sites that contain driver, modulator, and passenger residues rather than the more prevalent bystander residues (i.e. residues that do not mediate active state–specific contacts and are not important for pharmacology). Although the active state–specific contacts mediated by the passenger residues might minimize entropic cost by stabilizing a specific receptor conformation, thereby energetically favoring allosteric ligand binding, the perturbation of the driver or modulator residues at such sites might affect the allosteric network and hence mediate the positive or negative cooperativity at the orthosteric ligand binding site. In other words, a combination of (i) conformational stabilization of the active state by the allosteric ligand through interactions with passenger residues and (ii) perturbation of the allosteric network through interactions with surface exposed modulator or driver residues might contribute to the property of the allosteric ligand. An important implication of this observation is that the discovery of such surface-exposed driver, modulator, and passenger residues through the integration of pharmacological and structural data (in the presence of orthosteric ligands and absence of allosteric compounds) may reveal sites that could be targeted for development of novel allosteric ligands.

### Passenger, modulator, and driver residues are under higher evolutionary selection pressure

To assess the importance of the residues relevant for pharmacology and/or active-state conformation, we analyzed data on natural genetic variation from the human population. An analysis of the genome sequence data from over 140,000 individuals available through the gnomAD database (45) revealed a striking pattern in the distribution of single nucleotide polymorphisms (SNP) according to the different residue categories (Fig. 6D-E). Bystander residues harbored a larger number of positions with SNPs than what is expected by chance. Passenger and modulator residues contained fewer positions with SNPs than expected by chance. The allosteric network mediated by the connected driver residues had the lowest number of positions harboring SNPs in the human population, indicating that these residues were under the strongest selection pressure (Fig. 6D-E; **Methods**). Thus, driver, modulator, and passenger residues appear to be under differential selection pressure compared to bystander residues of this receptor in the human population, which is consistent with the importance of the residue categories.

To assess the importance of the residues across orthologs of adrenergic receptors from different species, we performed an analysis of Evolutionary Trace (ET) scores in the context of our residue classification (driver, modulator, passenger, bystander). ET is a phylogenetic method that identifies functionally important positions in protein families. Low ET scores indicate positions that are highly conserved across homologs. Depending on how the alignments are constructed, residues with low ET scores can also represent positions that are conserved in a sub-family-specific manner (46–48). Driver residues were the most conserved, followed by modulator, passenger, and bystander residues (Fig. 6F). Not all highly conserved residues were pharmacologically or structurally important; hence, the role of a residue cannot be predicted from evolutionary conservation alone. These findings reveal that categorizing residues based on structural and pharmacological importance can reveal category-specific associations of positions under purifying selection within the adrenergic receptors, aminergic receptors as well as the entire family of class A GPCRs (Fig. 6F, Fig. S13).

Mapping of the ET scores (adrenergic GPCRs) onto the allosteric networks revealed that driver residues with the lowest ET scores were at the center of the network in the receptor core, including all the network residues in TM3 and TM7 (Fig. S13). In contrast, the network positions with the lowest conservation (i.e., higher variability; ET scores > 3rd quartile, higher than 75% of the values) were either close to the ligand-binding site or in the intracellular region (Fig. S13). These positions are more tolerant to mutations and may evolve or have evolved to recognize new ligands or different G proteins (Fig. S13). This suggests that although a major part of the residues constituting the allosteric network is conserved within adrenergic GPCRs, key positions that interface with the ligand or G protein that are necessarily receptor-specific tend to be more variable. The uncoupling of ligand and G protein binding sites might have facilitated the evolution of GPCRs to respond to chemically diverse ligands and to couple to different G proteins.

Collectively, the observations on human genetics and evolutionary data, which are independent of the approaches we used to identify the residue classes, represent further evidence to support the model of the residues that govern the molecular origins of efficacy and potency in this signaling system.

## Discussion

Ligand efficacy and potency at a receptor are the key pharmacological parameters used to describe ligand-mediated receptor signaling. Although it is widely established that agonist binding activates the receptor and mediates structural rearrangements, ultimately resulting in a signaling response, we do not fully understand the molecular origins of this process and how each amino acid in the receptor decodes the information encoded in ligand atoms and collectively translates this into the emergence of efficacy and potency. We designed an integrative approach, combining pharmacological, structural, computational, and evolutionary analyses at a per-residue level, that revealed how receptors decode the information on the ligand atoms, mediate a series of structural changes through an allosteric network, and ultimately influence G protein binding and the downstream signaling response.

One-fifth of all residues in β2AR were important for adrenaline efficacy and potency for Gs signaling. Although some residues mapped to functionally important sites, such as ligand binding, G protein binding, and conserved motif residues, over two-thirds of the pharmacologically important residues mapped to other sites on the receptor, including those on the receptor surface. A conceptual framework that combined structural and pharmacological data helped us to propose potential roles for the receptor residues and allowed us to distinguish key, pharmacologically important structural changes from those that are less pharmacologically relevant. This framework resulted in the identification of an allosteric network of non-covalent contacts (based on active state-specific structural changes) that are mediated by pharmacologically important residues from the extracellular to the intracellular side. Our classification of residues into drivers, modulators, passengers, and bystanders provides a rational framework to explain the molecular and structural origins of potency and efficacy.

The exact nature of the pharmacologically and structurally important residues, as well as the allosteric network, likely depends on the choice of ligand, receptor, and effector. We expect considerable overlap for closely related systems based on the evolutionary analyses, but it is likely that the residues and the network will consist of non-overlapping receptor positions that depend on the specific ligand and the effector, including the G protein sub-type.

In addition to revealing such networks, the detailed analysis of ligand atom contacts with receptor residues presented here can help better understand the role of individual atoms in eliciting a pharmacological response. It can also aid in ligand design by enabling the identification of chemical groups that can be modified to achieve a desired signaling response. Our observation that surface exposed driver, modulator, and passenger residues identified using the endogenous ligand are contacted by negative and positive allosteric modulators suggests that such sites should be prioritized in high-throughput virtual screening efforts for the discovery of potential allosteric modulators.

Apart from revealing general principles, the approach and the findings presented here can help understand how disrupting and engineering parts of the network could alter receptor output, thereby facilitating the design of receptors with desired signaling properties (49). It could also help understand how changes in receptor residues, such as SNPs or disease-related mutations, could influence signaling responses to endogenous agonists or GPCR drugs. The framework developed here should enable the investigation of such questions for diverse ligand-receptor-signaling systems by leveraging high-throughput approaches, such as deep mutational scanning, to pharmacologically characterize mutations and the increasing availability of new structures of ligand-receptor-signaling protein complexes. Furthermore, the framework to classify residues based on pharmacological and structural relevance can be applied to any functional readout and property describing conformational change. In this manner, the approach can be extended to biological systems beyond GPCRs.

## Materials and Methods

### Experimental methods

#### DNA constructs and mutagenesis

The design of the human β2-adrenergic receptor (β2AR) construct was based on the commercially available pSNAPf -ADRβ2 Control Plasmid (NEB) coding sequence, which consist of a N-terminal signal sequence, the SNAP-tag® and the receptor sequence. A BamHI restriction site was added between the SNAP tag and the receptor sequence, the receptor sequence was codon optimised. The wild-type receptor amino acid at position 16 was arginine. The two most common alleles at position 16 are arginine and glycine (as defined by the 1000 genomes project). The SNAP-β2AR was cloned into pcDNA3.1. BRET-based biosensor constructs for Gαs, Gβ1, and Gγ1 subunits were in pcDNA3.1. The Gs biosensor consisted of RlucII-Gαs, where Renilla luciferase (RlucII) was inserted after amino acid 67, unmodified human Gβ1, and GFP10-Gγ1.

β2AR mutants for positions 2-412 were generated as described (50). Amino acids other than alanine were replaced by alanine, native alanines were replaced with glycines. Briefly, primers were designed using the custom-made software AAScan (51) (available here: https://github.com/dmitryveprintsev/AAScan) and ordered from Integrated DNA Technologies (IDT) as 500 pmol DNA oligos in 96-well plates. The primer sequences are available in Table S3. Forward and reverse mutagenesis primers were used in separate PCR reactions together with one primer each, which annealed in the ampicillin resistance gene (CTCTTACTGTCATGCCATCCGTAAGATGC and GCATCTTACGGATGGCATGACAGTAAGAG). PCRs were run as touchdown PCRs in 20 μl final volume. Resulting half-vector fragments were combined, digested with 0.5 μl DpnI (NEB) for 1 h, cleaned up using ZR-96 DNA Clean & Concentrator-5 (Zymo Research), assembled by Gibson assembly using HiFi DNA assembly Master Mix (NEB) at 45°C for 1 h (52) and transformed using Mix & Go competent cells (Zymo Research). The resulting colonies were cultured in 5 ml LB medium in 24-well plates overnight. The DNA was purified using Qiaprep 96 Plus kits (Qiagen) and sent for sequencing (Bio Basic Inc. or in-house). Sequences were analysed using custom software MutantChecker (available here: https://github.com/dmitryveprintsev/AAScan). Mutants that were not successfully generated as described were cloned using an alternative method: pcDNA3.1 was digested with NheI and XhoI (NEB), the β2AR coding sequence was amplified in two PCRs, each using one of the mutagenesis primers and either T7long or BGH primer (CGAAATTAATACGACTCACTATAGGGAGACCCAAGCTGG and TAGAAGGCACAGTCGAGG) which anneal upstream and downstream of the coding sequence, respectively. The two fragments were combined, digested using DpnI and cleaned up as described above. The clean fragments were assembled with digested, cleaned pcDNA3.1 using HiFi DNA assembly Master Mix (NEB).

#### Cell-culture and BRET-based signaling assays

Adherent HEK-293 SL cells (a gift from Stéphane Laporte) were cultured in DMEM with 4.5 g/l glucose, L-glutamine, supplemented with 10% newborn calf serum (NCS, Wisent BioProducts, Canada) and 1x penicillin-streptomycin (PS, 100X, Wisent BioProducts, Canada) at 37°C with 5% CO_2_. For transfections, DMEM without phenol red was used. Cells were transiently transfected using linear 25 kDa polyethyleneimine (PEI, Polysciences Inc., Canada, No. 23966) in a 3:1 ratio with DNA. Per condition, a total of 1 μg DNA was used to transfect 240’000 cells in 1.2 ml in suspension. The DNA mix consisted of 100 ng receptor DNA, combined with 75 ng Gαs-RlucII, 200 ng GFP10-Gγ1 and 100 ng Gβ1 plasmid DNAs; finally, 525 ng salmon sperm DNA (ssDNA) was added to bring the total amount of DNA to 1000 ng total in a volume of 100 μl. DNA was then combined with diluted PEI in phosphate-buffered saline (PBS) and added to the cells. Cells were seeded at 20’000 cells per well into Cellstar® PS 96-well cell culture plates (Greiner Bio-One, Germany) and incubated for two days at 37°C, 5% CO_2_. A Fluent780 liquid handler equipped with an MCA 96-well head (Tecan) and a Multidrop Combi (Thermo), each installed in a class II biosafety cabinet were employed to aid with transfections.

Before measurement, the medium was removed from the 96-well plates followed by the addition of Tyrode’s buffer (137 mM NaCl, 0.9 mM KCl, 1 mM MgCl_2_, 11.9 mM NaHCO_3_, 3.6 mM NaH_2_PO_4_, 25 mM Hepes, 5.5 mM glucose, 1 mM CaCl_2_, pH 7.4) and incubated for at least 30 min at 37°C. Plates were treated with ligand or vehicle (buffer control) for 10 min before measurement and with coelenterazine 400a (Nanolight Technology) at 5 μM final 5 min prior to measurement. Ligand concentrations used were: 31.6 nM (10^-8.5^ M) to 3.16 mM (10^-3.5^M) in half-log steps, diluted in vehicle, plus vehicle itself. Coelenterazine 400 a was prepared with 1% Pluronic F-127 in Tyrode’s buffer to increase solubility. Adrenaline was prepared in 0.01 M HCl to increase stability, the low pH was buffered by Tyrode’s buffer upon addition of ligand to the cells. The stability of adrenaline in the above aqueous solution was confirmed by comparing the signaling responses obtained immediately after ligand preparation, after 3h, 6h 20 min, 8h 40 min and 11 h. The BRET signal was read using a Synergy Neo (Biotek) equipped with dual photomultiplier tubes (PMTs, emission: 410 nm and 515 nm, gain 150 for each PMT and 1.2 s integration time). BRET was measured on an integrated platform with a Biomek NX liquid handler equipped with a 96-well head (Beckman, used for ligand addition), a multidrop combi (Thermo, luciferase substrate addition), an automated CO_2_ incubator Cytomat 6001 (Thermo, plate storage before experiment and during incubation), a barcode reader and Synergy Neo plate reader (Biotek). Ligand dilutions were kept at 4°C with the aid of a Peltier element. All signaling experiments were run in three biological replicates by default. In those cases where very low luciferase counts for multiple plates were observed, indicative of a failing transfection, all transfections and measurements of the day were repeated. All transfections and measurements for the days where an instrument failure led to a premature stop of the robotic system were repeated. BRET data for transfections with very low luciferase counts were completely removed during the data analysis (see manual curation).

All signaling experiments were carried out in at least biological triplicates, wild type and mock transfection controls were repeated six times per measurement day.

#### Cell-surface enzyme-linked immunosorbent assay (ELISA)

HEK-293 SL cells were transfected using PEI as above and seeded into poly-L-lysine coated 96-well plates (Greiner BioOne, Germany) and incubated for 2 days at 37°C 5% CO_2_. Each well was washed with 200 μl PBS, the cells were then treated with 50 μl 3% paraformaldehyde per well for 10 min. Each well was washed as follows: Three washes with wash buffer total (PBS + 0.5% BSA); for the last wash step, the cells were incubated with wash buffer for 10 min. Primary rabbit anti-SNAP antibody (GenScript, USA) was added at 0.25 μg/ml in 50 μl and incubated for 1 h at RT, followed by three wash steps as described above. The cells were incubated with secondary anti-rabbit HRP antibody (GE Healthcare) diluted 1:1000 for 1 h. Again, the cells were washed three times with wash buffer and then three times with PBS. Per well, 100 μl SigmaFast solution was added, the plates were incubated at RT in the dark. Reactions were stopped by the addition of 25 μl 3M HCl. 100 μl of the resulting solution was transferred to a new transparent 96-well plate, and the optical density was read at 492 nm using a Tecan GENios Plus microplate reader. Cell-surface ELISAs were carried out in three biological replicates with internal quadruplicates. Each plate contained wild-type and mock-transfected cells as controls.

#### Analysis of biosensor signal dependence on cell-surface expression

Twelve different amounts of wild-type receptor DNA were combined with constant amounts of biosensor DNA and transfected into HEK293 cells as described above. The amounts of wild-type receptor DNA were 0.05, 0.1, 0.2, 0.5, 1, 2, 5, 10, 20, 50, 150 and 200 ng. Two days after transfection, the cell-surface expression level and Gs biosensor BRET signal were determined for each transfection. Cell-surface expression in percent of the level obtained for a 100 ng wild-type receptor DNA transfection (the standard used for signaling experiments as described above) was then plotted against Gs biosensor signal amplitude and Gs biosensor logEC50 (Fig S3A-B). All experiments were repeated in three to four biological replicates.

### Computational methods & data analysis

#### Processing of signaling data

The BRET data were processed using BRET2DTF and DataFitter custom software (Dmitry Veprintsev; https://github.com/dbv123w/DataFitter). All biological replicates of concentration-response curves were separately fitted to a Hill equation using a Hill slope of 1 to determine the agonist concentration at the half-maximal signal response (logEC50) as well as the pre-transition and post-transition baselines (may also be referred to as “top” and “bottom”). All concentration-response curves were visually inspected. We removed complete repeats (i.e., all twelve data points) in those cases where the experiment had obviously failed due to a transfection that did not work (see explanation in “BRET-based signaling assays” above) or where data could not be fitted reliably due to noise. In the whole mutant data set of 1324 concentration-response curves (number excludes controls), 42 single replicate curves were excluded. In addition, replicates for 7 mutations showed no measurable signaling (see Table S1, curation column set to 2). Data could be fitted starting at approx. 20% of wild-type amplitude, corresponding to approx. 0.015 difference between the pre-and post-transition baselines in raw BRET (where raw BRET = GFP/RlucII signal). In total, after these curation steps, quadruplicates for 77 mutants, triplicates for 306 mutants, and duplicates for 29 mutants were retained. No single data points were removed in any case.

The results from data fitting were read into R (RStudio 1.3.959 to 2022.07.1 and R 4.0.0 to 4.2.1) and processed using custom scripts. The following packages were used: tidyverse (especially dplyr, ggplot2, purrr, tibble, tidyr, forcats, stringr), plotly, MASS, reshape, reshape2, ggrepel, patchwork, ggpubr, bio3d (53), openxlsx.

The data were read in, reformatted, averaged, annotated (addition of GPCRdb number, expression level, amino acid number, mutation), filtered based on the results of manual curation, normalized, and visualized in R. We checked the data set for outliers, day-to-day variation and trends, the effect of expression level on signaling response and baseline signal variation. We compared the BRET signal for mock transfections where ssDNA was used instead of receptor DNA with mutant pre-transition baselines to assess possible changes in constitutive activity. Based on the variability of wild-type pre-transition baselines we decided not to interpret changes in mutant pre-transition baselines. While we were not able to measure changes in constitutive activity reliably, this does not mean that none of the mutants affect constitutive activity. Measured mutant amplitudes were normalized to WT using the most recently measured wild type data set to correct for day-to-day variation and variation during the day, while mutant logEC50s were normalized to the mean logEC50 obtained for wild type. The normalization did not correct for expression level since the dependence of the signal was minor for receptor expressing to at least 25% of wild-type level, see Fig. S3A-B. The measured logEC50 and amplitude values were also used to interpret 22 receptor positions in Hauser *et al*. (15) and Hedderich *et al*. (44). The typical error of an ELISA was much larger than the error of a BRET experiment, at least in our experience; such a correction would therefore have rather lowered the confidence in the results. Cut-offs were applied to the normalized, and curated data, resulting in discretization. Once normalised, the wild-type data were centred around a normalised amplitude of 1 and a normalised logEC50 of 0. Cut-offs were chosen so that none of the 57 wild-type concentration-response curves (measured on different days and batches) were classified as not wild-type like. For the normalized amplitude, the cut-off was < 0.74 (this corresponds to two wild-type standard deviations). For the normalized logEC50, the cut-off was > 0.87, this corresponds to a 7.4-fold change.

Our cut-off for a gain of function would be > 1.28 for the normalized amplitude and < -0.71 for the normalized logEC50 (cut-offs were applied to non-rounded values; Table S1 values are displayed to two decimal precision). As we were interested in identifying those positions whose mutation impairs signaling, the one mutation that increased efficacy was not explicitly considered in our analyses.

Graphs and illustrations were generated in R (ggplot2), Adobe Illustrator CS6 and CC and Affinity Designer 2. For display in figures, concentration-response curves were fitted in R using the package drc. The raw BRET values were fitted to obtain the BRET value for the pre-transition baseline. Subsequently, the data were normalised using the pre-transition baseline and the wild-type signal amplitude, resulting in a normalised BRET response where 0% and 100% correspond to the wild-type response. For fitting of the normalised data, the LL.4 function was used, the slope was fixed to 1 and the pre-transition baseline was fixed to 0.

#### Residue-residue contacts

Residue-residue contacts were obtained from Protein Contacts Atlas (http://pca.mbgroup.bio/index.html) (54). Two residues are listed as a contact if the distance between the two atoms minus their van der Waals radii equals 0.5 Å or less, corresponding to a maximum distance of ~4.2 Å. Text files downloaded from Protein Contacts Atlas containing residue-residue contacts were read into R and cleaned. We then fortified the tables with additional information (secondary structure elements (SSE) and GPCRdb numbers for each amino acid), filtered the contact tables to exclude main chain-main chain contacts, and include contacts between two different SSEs only. The contacts were filtered to include only those residues resolved in both crystal structures used for the comparison, 2RH1 and 3SN6. In the inactive and in the active G protein-bound structures of the β2AR, 282 and 285 residues form 1275 and 1233 residue-residue contacts, respectively. To focus on residue contacts that contribute to large domain motions instead of local rearrangements during activation, we excluded contacts formed exclusively between backbone atoms and contacts within the same secondary structure element (i.e., contacts formed within a single helix). This reduced the number of residue-residue contacts to 117 unique contacts in the inactive state, 61 unique contacts in the active, G protein-bound state, and 191 contacts that are present in both the active and inactive states. Contacts are listed in Table S2.

#### Definition of the ligand and G protein-binding sites

Definitions of the ligand-binding site and the G protein-binding site were based on the structures of β2AR with adrenaline and an engineered nanobody (PDB 4LDO) (25), in complex with heterotrimeric G protein and Gs peptide (3SN6 and 6E67) (35) (55). All residues within 4 Å of adrenaline were classified as part of the ligand-binding site, the residues were verified using LigPlot+ v.2.2 and literature data (26, 56) (57) (58) (59). Using PyMOL 2.5.2, all residues within 4 Å of G protein or Gs peptide were determined and classified as residues in the G protein-binding site. Additionally, this residue selection was confirmed by atom-atom contacts from the Protein Contact Atlas. Residues in the ligand and G protein-binding site, respectively, are marked in Table S1.

#### Residue classification

We defined driver and modulator residues as those residues that affect Gs signaling upon mutation (see above for cut-offs). Additionally, disconnected drivers participate in an active-state specific contact and connected driver residues form an active-state specific contact to another driver residue. In addition, both connected driver residues need to negatively affect potency, or both need to affect efficacy. That is, if the mutation at one driver residue affected potency and the mutation at the interacting driver residue affected efficacy they were classified as disconnected drivers due to a mismatch in effects. Passenger and bystander residues were defined as those residues that do not affect Gs signaling upon mutation. Additionally, passenger residues must form at least one active-state specific contact. Residues not forming such contacts and not affecting Gs signaling upon mutation were classified as bystanders. Hierarchically, we defined (a) connected drivers as the structurally and pharmacologically most important residues, followed by (b) disconnected drivers and (c) modulators that are pharmacologically important but do not mediate active-state specific contact, (d) passenger residues which mediate active-state specific contact(s) but don’t affect pharmacology, and finally (e) bystanders, which are not involved in forming an active-state specific contact and do not affect pharmacology. The allosteric network consisted of 15 residue-residue contacts (33 atom-atom contacts) between 23 residues. Of the 33 atom-atom contacts, 26 were side chain-side chain contacts and 7 were main chain-side chain contacts.

#### Evolutionary Trace analysis

We used Evolutionary Trace Analysis to identify β2AR residues with evolutionarily encoded functional roles at the cross-section of the network of pharmacologically important residue positions. Evolutionary Trace scores (ET scores) were obtained from the Lichtarge Lab Evolutionary Trace webserver (http://evolution.lichtargelab.org/gpcr. We compared the conservation of amino acids at the level of the adrenergic receptor subfamily (“Adrenoceptor (118)”), amine receptors (“All_amine (547)”), and class A (“ClassA_5105”) GPCRs. For each phylogenetic comparison, the lower ET scores at a residue position represent high conservation of specific amino acids at the position within the phylogenetic class. We compared the enrichment of low ET score positions amongst the residues found in the “driver”, “modulator”, “passenger”, and “bystander” categories. Groups were compared using a Wilcoxon test (* p ≤ 0.05, ** p ≤ 0.01, *** p ≤ 0.001, **** p ≤ 0.0001, ns: non-significant).

#### Extraction of structure features

To calculate the distance between Cα atoms of each residue in the inactive versus the active state, we used the highest-resolution inactive-state structure (PDB ID: 2RH1) and the only available G protein complex structure at the time of performing this analysis (PDB ID: 3SN6) of the β2AR, aligned the two structures in PyMOL and exported the new coordinates. The pdb files were read into R using the package bio3d, followed by extraction of the x, y, and z coordinates for each structure and the calculation of distance as:

$$distance=\sqrt{{\left(x_{active}-x_{inactive}\right)}^{2}+{\left(y_{active}-y_{inactive}\right)}^{2}+{\left(z_{active}-z_{inactive}\right)}^{2}}$$

Likewise, we extracted the dihedral angles for each residue in the above structures using bio3d. The difference in the angles ϕ, ψ and ω between active and inactive state was calculated as:

$$\text{Δ}\phi =(\phi_{active}-\phi_{inactive}+180)\;\%\;360-180$$

#### Calculation of accessible surface area

Accessible surface area was calculated using dssp (https://swift.cmbi.umcn.nl/gv/dssp/) (60, 61) and values are provided in Table S1. In addition, we calculated the accessible surface area of the adrenaline binding site with and without the ligand present using MDTraj (https://www.mdtraj.org) and GROMACS (https://www.gromacs.org/).

#### Analysis of single-nucleotide polymorphism data

Single-nucleotide polymorphism (SNP) data for ADRB2 was retrieved from the Genome Aggregation Database (gnomAD, https://gnomad.broadinstitute.org/, version gnomAD v2.1.1). The data were analysed using custom scripts with functions from the tidyverse (especially readr, dplyr, ggplot2), and the packages janitor, rebus, and stats. Statistical over- and underrepresentation of SNPs in driver, modulator, passenger, and bystander positions was calculated using a hypergeometric test.

## Supplementary Material

Supplementary Information

Supplementary Material Table Captions

Table S1

Table S2

Table S3

## Figures and Tables

**Figure:**
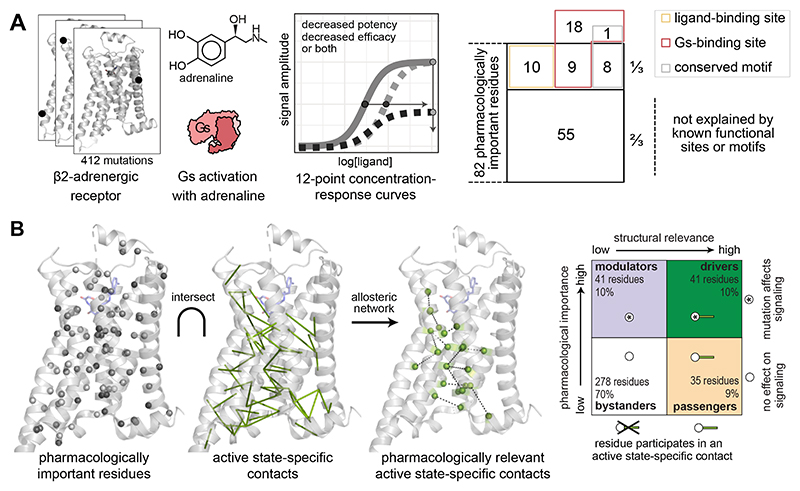


## Data Availability

The datasets generated during and/or analyzed during the current study are available in Table S1 and Table S2. The sequences of the primers used for mutagenesis are listed in Table S3. All data are available in the main text or the supplementary materials; code can be accessed at https://doi.org/10.5281/zenodo.8349360 or https://github.com/mb-group/DeterminantsSignalingGPCR.
