# Supplementary material for: Molecular determinants of ligand efficacy and potency in GPCR signaling
Source: Science. Author manuscript; Available in PMC 2024 Jan 16. (PMC7615523; doi:10.1126/science.adh1859)
Supplement: Supplementary Information [file EMS193248-supplement-Supplementary_Information.pdf]

Science
AAAS

Supplementary Materials for

Molecular determinants of ligand efficacy and potency in GPCR signaling

Franziska M. Heydenreich[1,2,4,$,*], Maria Marti-Solano[2,3 †], Manbir Sandhu[4, †], Brian K. Kobilka[1,*], Michel Bouvier[5,*], M. Madan Babu[2,4,*].

Correspondence to: franziska.heydenreich@pharmazie.uni-marburg.de, kobilka@stanford.edu, michel.bouvier@umontreal.ca; madan.babu@stjude.org

This PDF file includes:

Figs. S1 to S13

Other Supplementary Materials for this manuscript include the following:

Tables S1 to S3

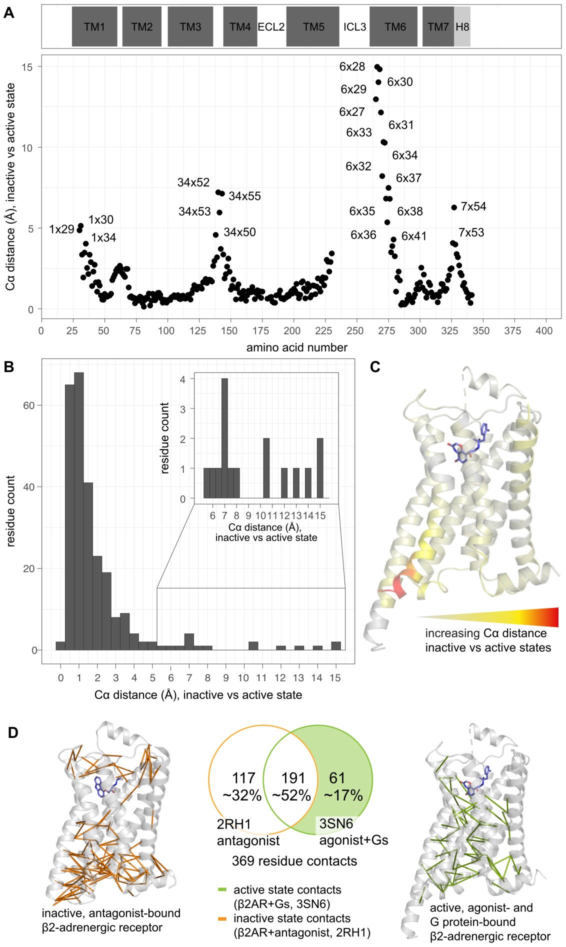

Fig. S1. Structural features of the β2-adrenergic receptor. (**A**) Distance between Cα atoms in the inactive versus active states (PDB IDs: 2RH1 and 3SN6) by amino acid number. The top row depicts secondary structure elements (TM = transmembrane helix, ECL = extracellular loop, ICL = intracellular loop). Residues with a Cα movement over 5 Å between inactive and active states are labelled using their GPCRdb number. (**B**) Histogram of Cα distances moved, the inset provides a more detailed view of Cα distance distribution over 4 Å. (**C**) Structure of the β2AR in the active state (PDB ID 3SN6) coloured by the distance between Cα atoms in the inactive state (PDB ID 2RH1) versus the active, G protein-bound state (PDB ID 3SN6). (**D**) Non-covalent residue contacts specific to the inactive state (orange) and the active state (green) are displayed on the respective structures (2RH1 and 3SN6). Contacts between neighbouring residues in the same secondary structure element were not considered. The Venn diagram displays the number of inactive state-specific, shared, and active state-specific contacts. In the adrenaline-β2-adrenergic receptor (β2AR)- Gs signaling system, 11 residues contact the ligand, 28 residues contact the G proteins, 22 residues translocate over 4 Å during activation, and 81 residues mediate 61 active state-specific contacts. Are all residues involved in structural change important for signaling? If not, which residues are important and what roles do the different residues play in contributing to ligand efficacy and potency at the receptor?

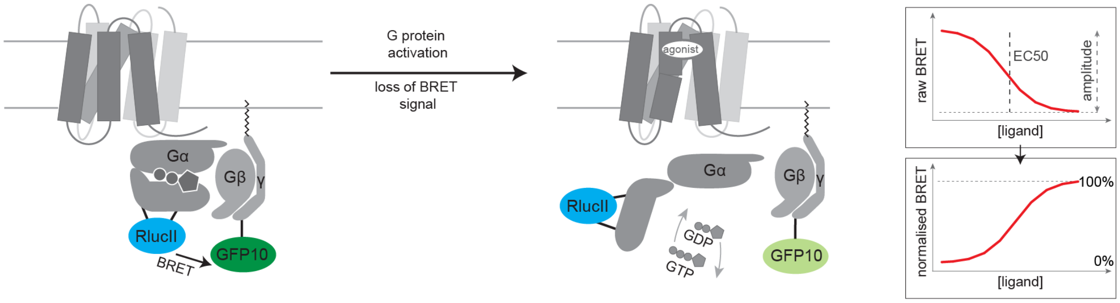

Fig. S2. BRET-based biosensor for Gs activation. To measure Gs activation in HEK cells, we used a BRET-based biosensor for Gs activation. The Gα subunit was fused with *Renilla reniformis* luciferase (RlucII, donor) and the Gγ subunit was labelled with GFP10 (acceptor). Upon G protein activation, the Gα subunit changes its conformation and dissociates from Gβγ, leading to a decrease in BRET signal. The raw values of BRET ratios are normalized to obtain the concentration-response curve and quantification of ligand efficacy and potency of adrenaline-stimulated β2AR activation (**Methods**).

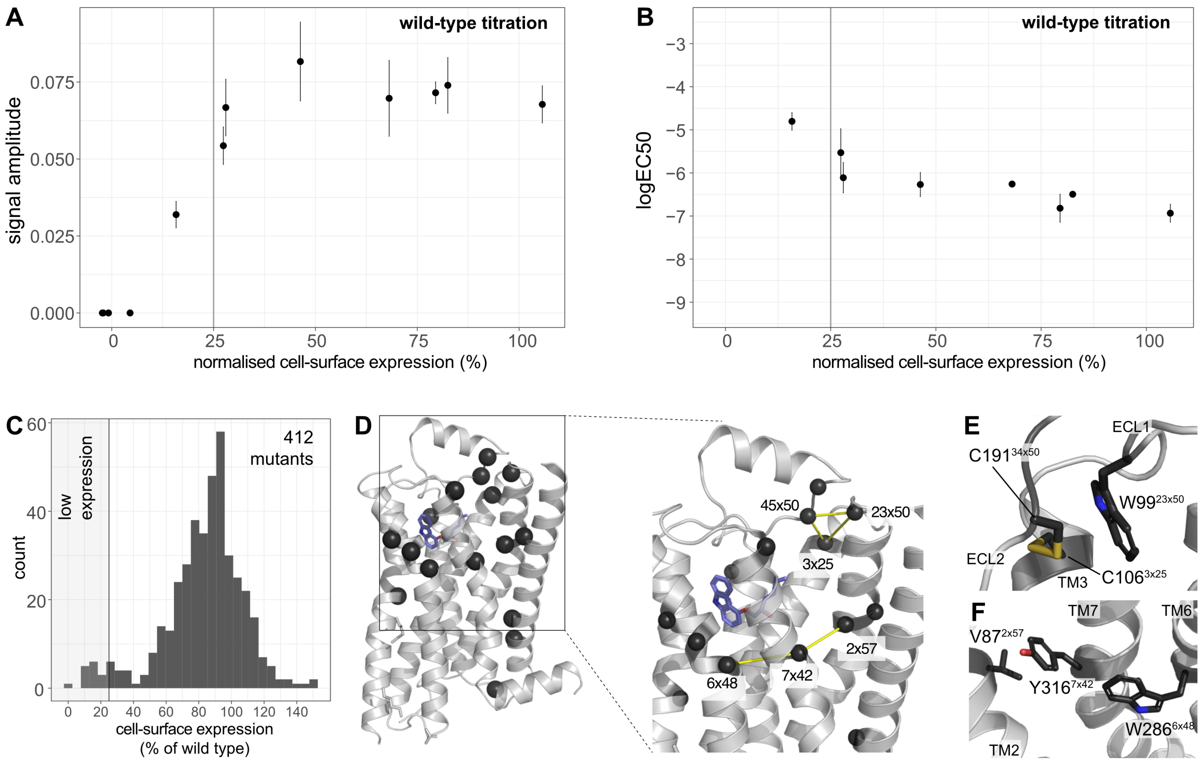

G

amino acid	mutation	GPCRdb	expression	adrenaline binding	G protein binding	location
51	N51A	1x50	18.8	0	0	core
55	I55A	1x54	14.5	0	0	core
64	L64A	12x50	17.0	0	0	other
87	V87A	2x57	11.2	0	0	core
88	P88A	2x58	14.3	0	0	core
99	W99A	23x50	9.2	0	0	other
106	C106A	3x25	15.7	0	0	core
168	P168A	4x60	11.6	0	0	core
190	C190A	ECL2	23.5	0	0	other
191	C191A	45x50	14.0	0	0	other
211	P211A	5x50	12.7	0	0	core
286	W286A	6x48	10.1	0	0	core
288	P288A	6x50	16.3	0	0	core
291	I291A	6x53	0	0	0	core
306	E306A	7x32	22.7	0	0	core
316	Y316A	7x42	19.0	1	0	ligand

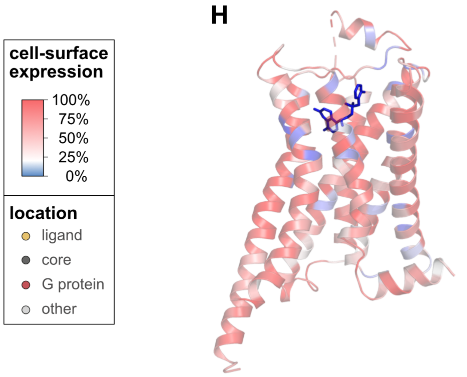

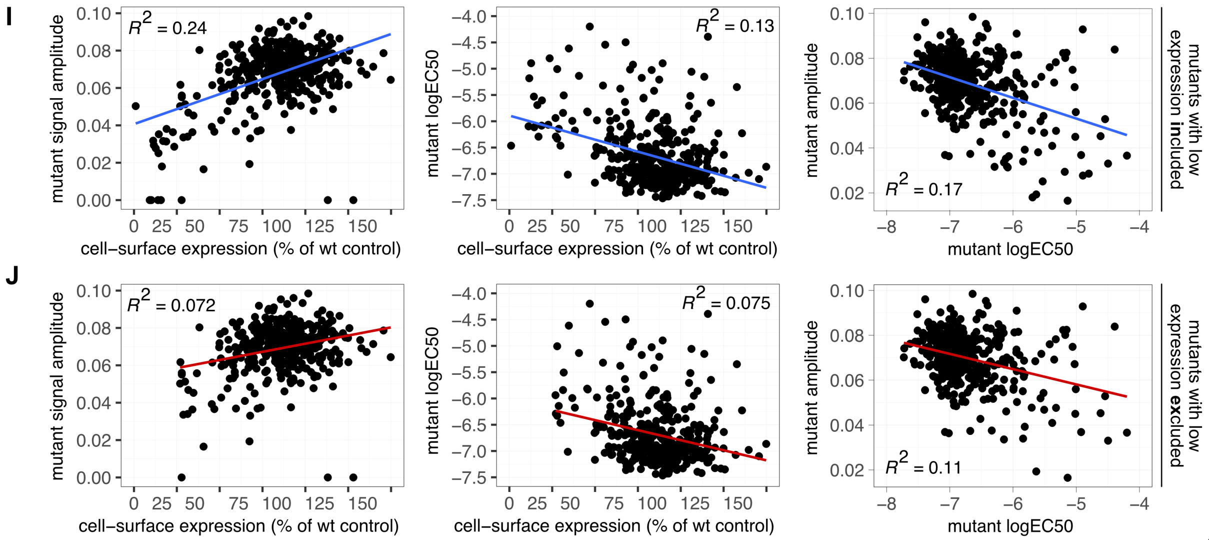

Fig. S3. Residues important for cell-surface abundance. Since low cell-surface abundance negatively influences signaling measurement, cell-surface ELISA was used to evaluate the expression levels of all mutants. (**A**) Calibration/titration curves of wild type receptor abundance against BRET signaling parameters. Variation of the Gs signal amplitude with cell-surface abundance for wild-type receptor, with SEM error bars from triplicate or quadruplicate measurements. Here, different amounts of WT DNA were transfected together with biosensor plasmids, followed by determination of signal amplitude, logEC50 and cell-surface abundance (**Methods**). Below 25% abundance (vertical line), the BRET signal strongly decreased. Therefore, we set the receptor abundance cut-off (for obtaining reliable measurements) at 25% of WT level. Thus, mutants with abundance <25% of the wild-type level (and any measurements of signaling properties) were excluded from pharmacological analysis. (**B**) Variation of the logEC50 with cell-surface abundance for wild-type receptor. (**C**) Cell-surface abundance of 412 single point mutations shown as a histogram. Low abundance was defined as <25% of wild-type level. (**D**) Positions of the 16 residues whose mutation led to a strong decrease in cell-surface abundance are shown as black spheres. Two residues mapped to the first TM helix ($N51^{1x50}$ and $I55^{1x54}$), suggesting that they could be critical for membrane protein folding and biogenesis in the endoplasmic reticulum (ER) membrane. One mutation mapped to the intracellular loop 1 (ICL1; $L64^{12x50}$), a residue previously identified as important for ER export (Duvernay *et al.*, *Traffic*, 2009). Six residues made two distinct sets of contacts with each other in the structure. The first set involved a conserved disulphide bridge and a neighbouring tryptophan ($C106^{3x25}$, $C191^{34x50}$, $W99^{23x50}$), recently shown to be particularly intolerant to mutation (Jones *et al.*, *eLife*, 2020)). The other connected helices 2, 6, and 7 ($V87^{2x57}$, $W286^{6x48}$, and $Y316^{7x42}$). (**E**) and (**F**) stick representation of the indicated networks. (**G**) 16 residues with low abundance as defined above (their abundance shown in % of WT abundance), and whether they are in the ligand or G protein binding sites (1) or not (0). (**H**) Cell-surface abundance of mutants, colour-coded and mapped to the 3D structure (PDB ID 3SN6). (**I**) Correlation between receptor abundance vs amplitude (left), abundance vs logEC50 (middle), and logEC50 vs amplitude (right) for all mutants regardless of cell-surface abundance. Mutants with no measurable signaling were excluded in those cases where logEC50 was plotted (middle, right) since no value could be determined (**J**) Correlation only for the 396 mutants for which receptor abundance was >= 25% of the WT receptor level (the set that was used for subsequent analyzes in this paper after applying the abundance cutoff). The removal of the 16 mutants with abundance <25% WT removes any weak correlation that existed in the data. As in (I), mutations with no measurable signaling were removed from the middle and right plots.

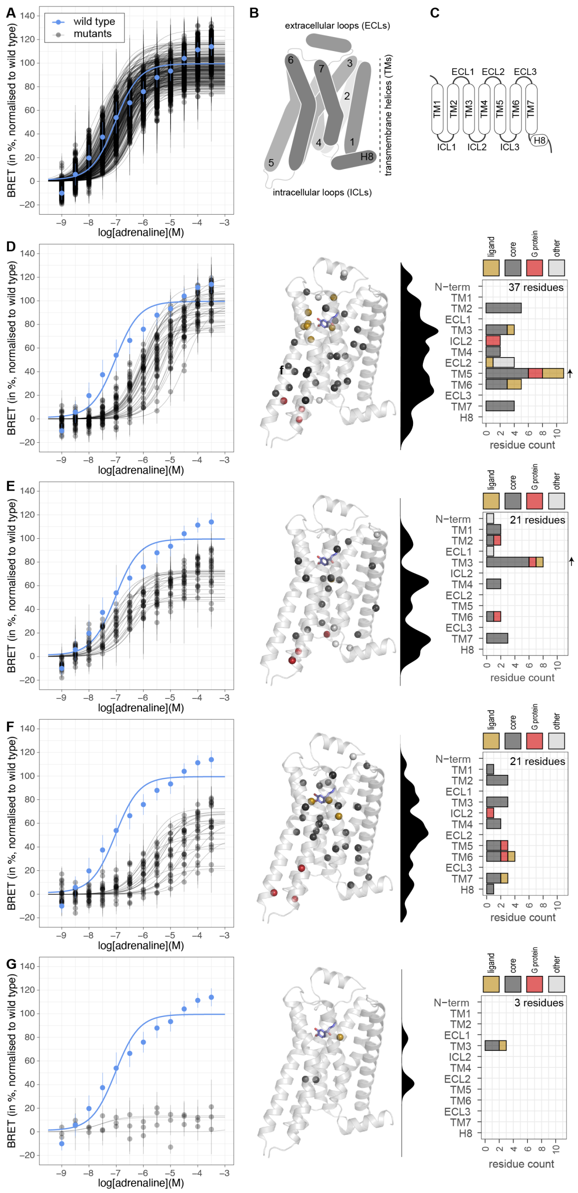

Fig. S4. **Concentration-response curves for mutants with different pharmacological effects.** (**A**) Overview of potency (normalized logEC50) and efficacy (normalized signal amplitude) of all β2AR mutations with wild-type-like signaling responses (black) and wild-type (blue). The data points of multiple independent measurements for the wild type were averaged and fitted for illustration purposes only (see **Methods**). (**B**, **C**) Cartoon representation of β2AR showing the position of secondary structure elements. Transmembrane helices are numbered. (**D-G**) Signaling profile, position of mutated residue on the structure (PDB 4LDO), the density of mutations on the extra- to intracellular axis and a bar graph of the location of mutations in relation to its secondary structure element and functional regions is shown for mutations with decreased potency (**D**), decreased efficacy (**E**), decreased potency and efficacy (**F**), and mutations that decreased signaling to a non-measurable level (but still expressed) (**G**). Arrows in bar graphs of D and E indicate statistical overrepresentation of mutations with a certain effect in the secondary structure element (**Methods**). Ligand-binding pocket and G protein–binding sites were defined as residues within 4 Å of adrenaline (PBD 4LDO) or Gs (PDB 3SN6).

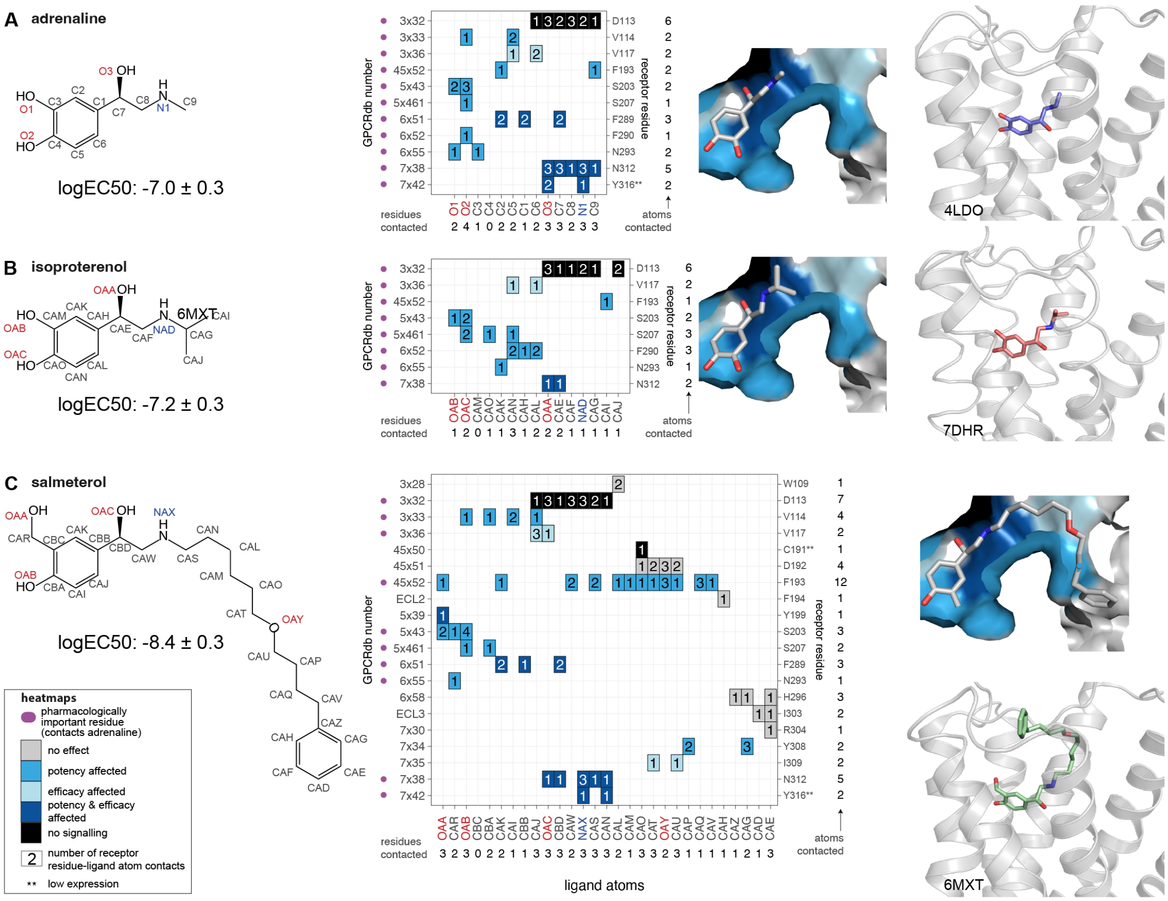

Fig. S5. Comparison of ligand atom - receptor residue interactions for adrenaline, isoproterenol, and salmeterol. Comparison of (**A**) adrenaline, (**B**) isoproterenol, and (**C**) salmeterol chemical structures (left), contacts formed by each ligand atom with receptor residues (middle), surface of the ligand binding site colored by pharmacological effect of the mutation on Gs signaling in response to adrenaline as the ligand (right) and cartoon-and-stick representation of ligand binding to the receptor [PDB IDs: 4LDO (adrenaline), 7DHR (isoproterenol) and 6MXT (salmeterol)]. F193 contacts two adrenaline atoms and affects potency only when mutated. This suggests that chemical modification of ligand atoms that more extensively interact with such a residue could allow for targeted modification of potency response at the receptor, which is the case with F193 and salmeterol (a ligand with higher potency).

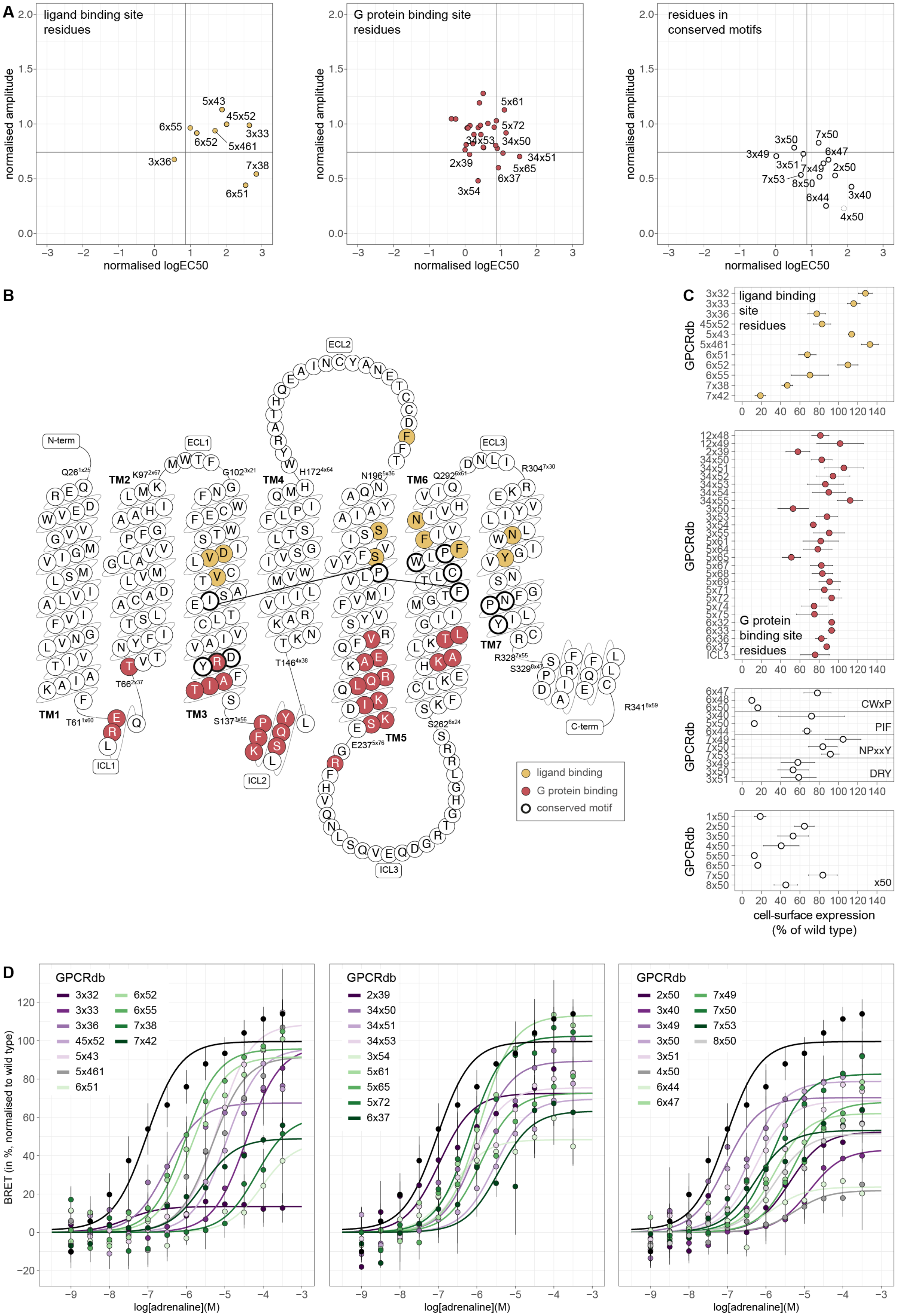
A
ligand binding site residues
G protein binding site residues
residues in conserved motifs
normalised amplitude
normalised logEC50
5x43
45x52
6x55
3x33
6x52
5x461
3x36
7x38
6x51
5x61
5x72
34x53
34x50
34x51
2x39
5x65
6x37
3x54
3x50
7x50
3x49
6x47
3x51
7x49
2x50
7x53
8x50
3x40
6x44
4x50
B
N-term
ECL1
ECL2
ECL3
ICL1
ICL2
ICL3
C-term
TM1
TM2
TM3
TM4
TM5
TM6
TM7
ligand binding
G protein binding
conserved motif
C
ligand binding site residues
G protein binding site residues
GPCRdb
CWxP
PIF
NPxxY
DRY
x50
cell-surface expression (% of wild type)
D
GPCRdb
BRET (in %, normalised to wild type)
log[adrenaline](M)

Fig. S6. Pharmacological importance and cell-surface abundance of mutants mapping to binding sites and conserved motifs. (**A**) Pharmacological effect of mutations in the ligand binding site (left, yellow circles), the G protein binding site (middle, red circles) and conserved motifs (right, white circles). The x-axis shows normalized logEC50, (higher numbers indicate a loss in potency), and the y-axis shows normalized signal amplitude (lower numbers indicate a loss in efficacy). Mutations with a pharmacological effect are labelled with their GPCRdb number. (**B**) Snake plot showing the positions of residues in the ligand binding site (yellow), the G protein binding site (red) and conserved motifs (bold outline). Residues of the PIF motif are connected by lines. (**C**) Relative cell-surface abundance of alanine or glycine mutants in the ligand-binding site (yellow, top), the G protein binding site (red, middle), conserved motifs (white, middle) and the most conserved residue in each helix (white, bottom). (**D**) Concentration-response-curves of all ligand-binding site (left), G protein binding site (middle) and conserved motif residue (right) mutants for Gs with the ligand adrenaline. The data points of 57 independent measurements for the wild type were averaged and fitted for illustration purposes only (see **Methods**).

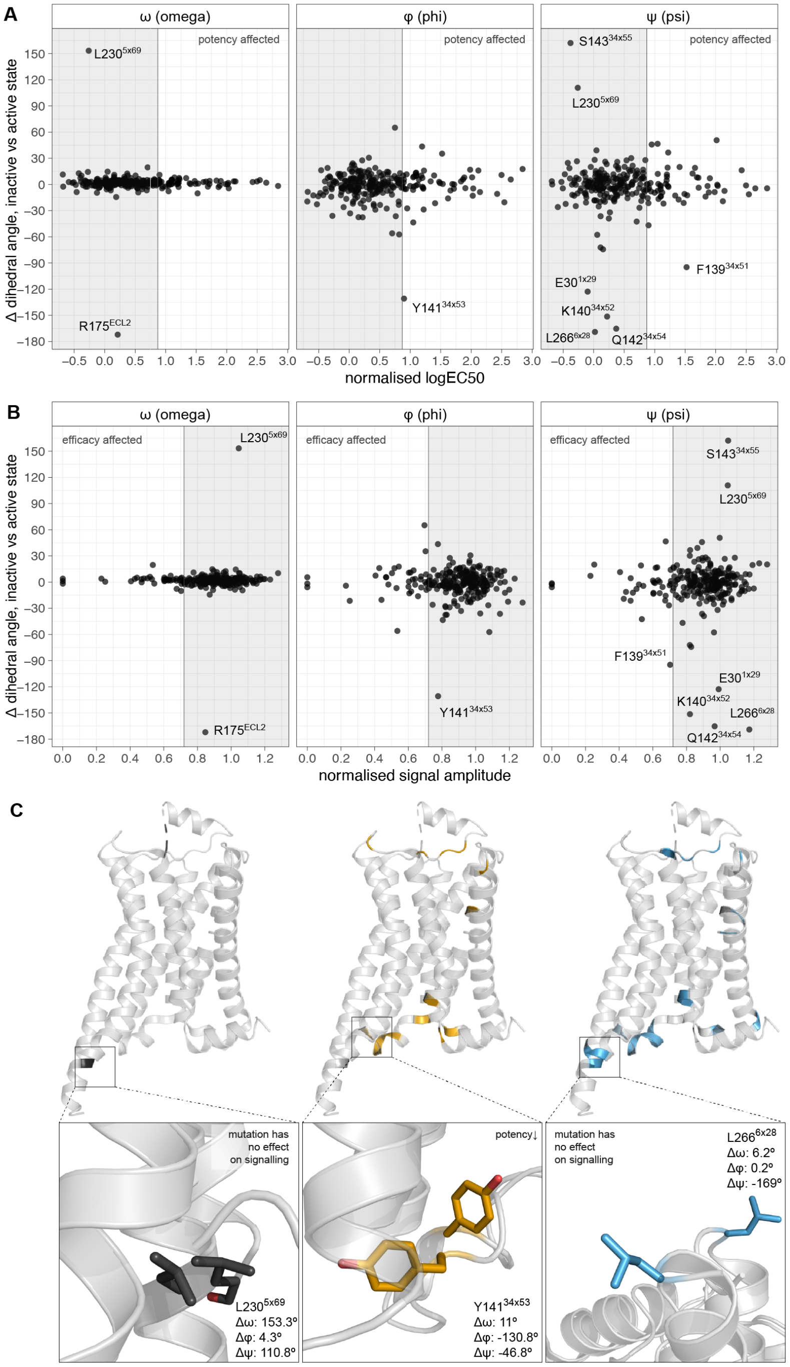

Fig. S7. Absence of association between dihedral angle change during activation and the pharmacological effect upon mutation. (**A**) Difference (delta) in dihedral angles for the inactive and active state structures (PDB IDs: 2RH1 and 3SN6), plotted against the normalized logEC50 (potency) for each corresponding mutation. (**B**) Delta dihedral angle plotted against the normalized signal amplitude (efficacy). (**C**) Three examples of positions with high changes in dihedral angle between inactive and active state with details shown for the residue. Residues with large dihedral angle changes show little effect upon mutation and residues with small dihedral angle changes show significant effect on pharmacology upon mutation.

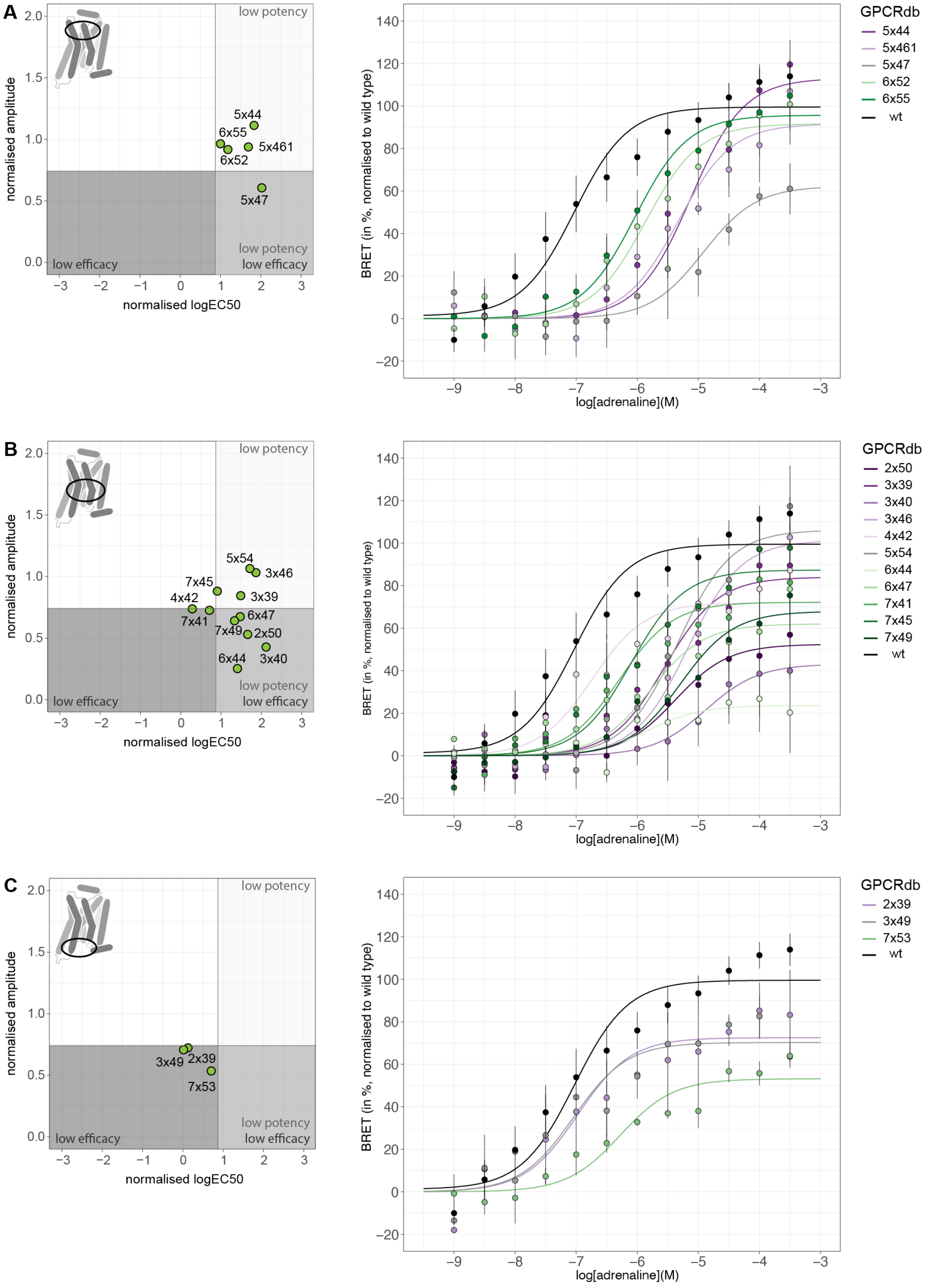

Fig. S8. Effect of mutating residues in the allosteric network on Gs activation. (**A**) Effect of mutating residues in the extracellular region, (**B**) network residues in the receptor core, and (**C**) network residues in the intracellular region (defined as any residue within 10 Å of G protein). The data points of multiple independent measurements for the wild type were averaged and fitted for illustration purposes only (see **Methods**).

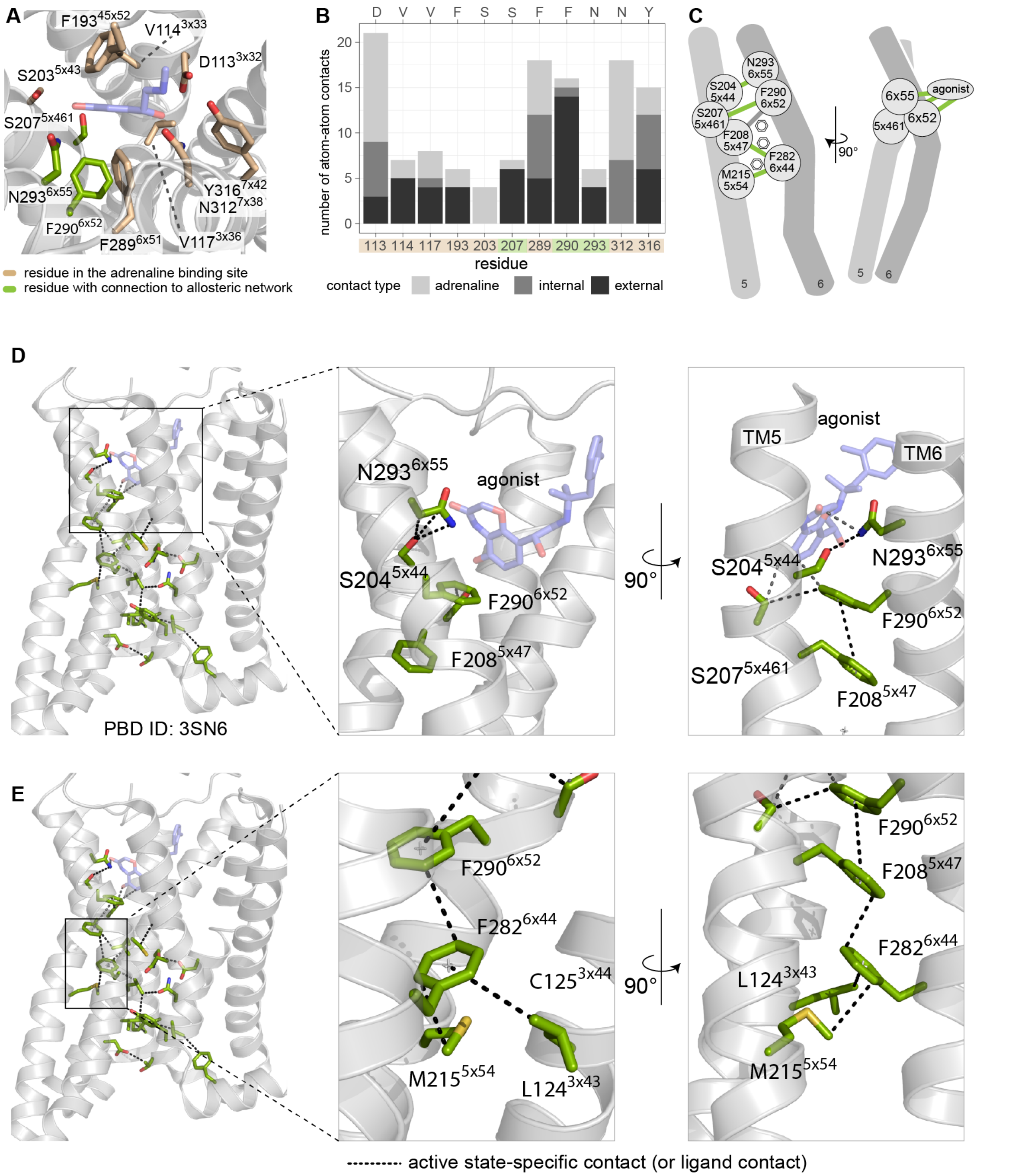

Fig. S9. Interaction of the ligand with residues in the allosteric network. (A) Structural representation of the adrenaline binding site (PDB 4LDO), interacting residues are shown as sticks. **(B)** Number of atom-atom contacts formed by atoms in binding site residues with the ligand or receptor residue atoms. Counts show the number of contacts formed with adrenaline atoms (light grey), other binding site residues (dark grey; internal), or residues outside of the binding site (black; external). **(C)** Residues connecting the ligand binding site with the allosteric network **(D and E)** detailed view of the extracellular side of the allosteric network.

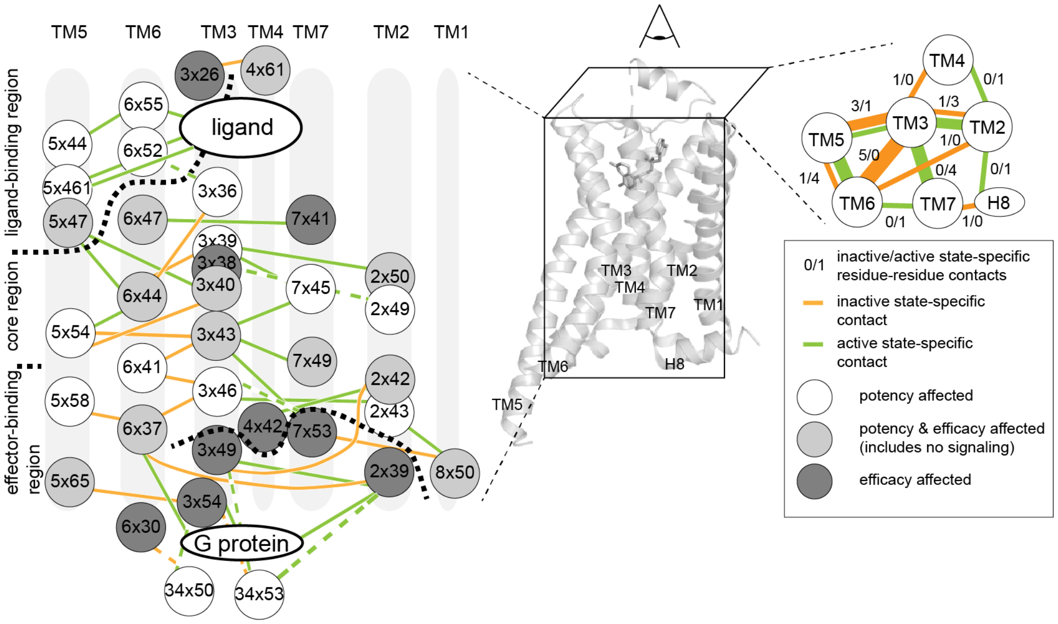

Fig. S10. Comparison of inactive state- and active state-specific residue contact networks. Left: orange lines indicate residue contacts that are specific for the inactive state (PDB ID: 2RH1) while green lines indicate residue contacts specific to the active state (PDB ID: 3SN6). Dashed green or orange lines indicate contacts between residues important for efficacy only and potency only, respectively, which were not included in the allosteric network for the activated receptor. Right: Top view of the receptor with the number of state-specific contacts formed between helices shown as inset.

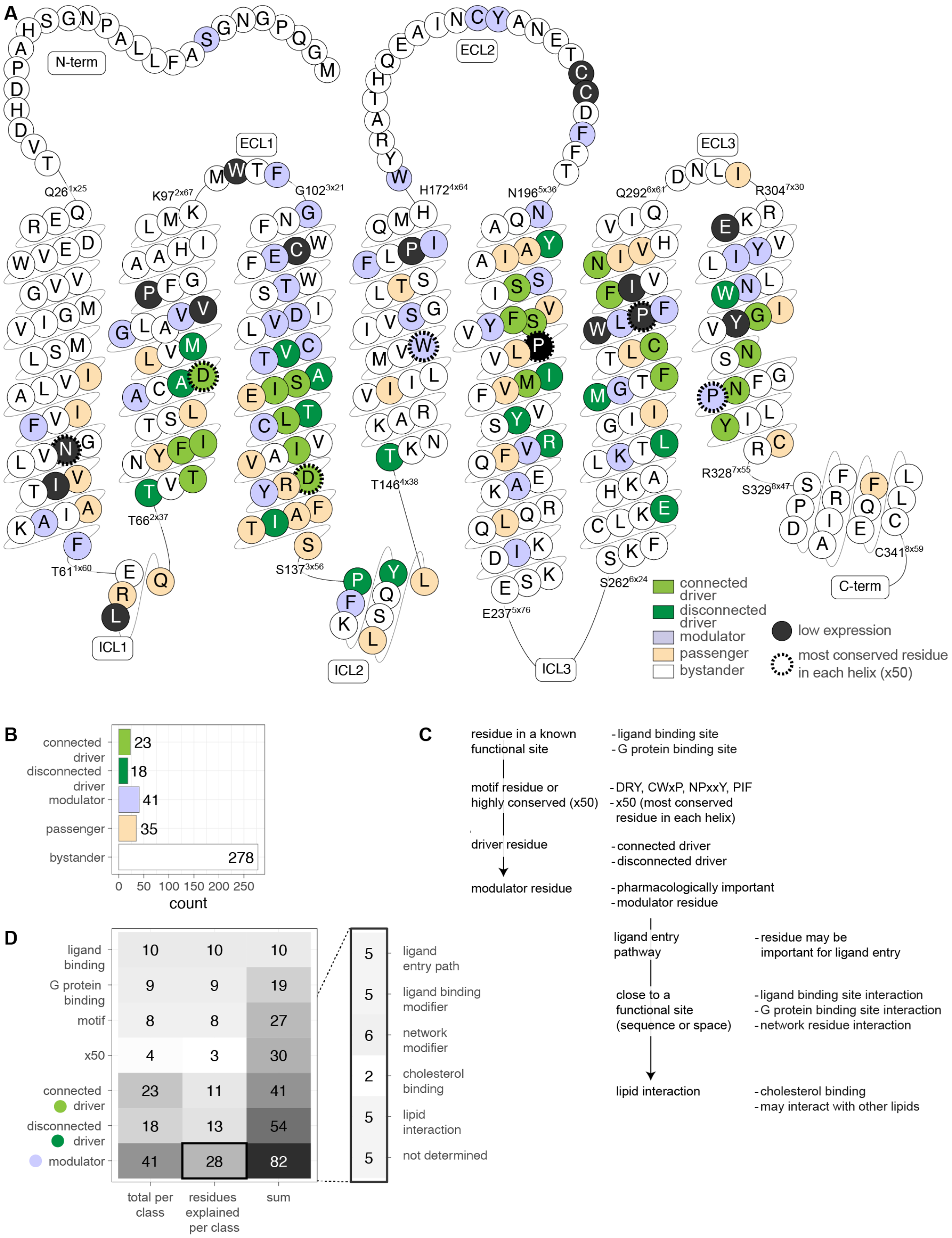

Fig. S11. Distribution and function of residues by structural and functional classification. (**A**) Snake plot of β2AR with residue coloured by structure-function classification (green: structurally and pharmacologically important drivers; slate: pharmacologically important modulators; wheat: structurally important passengers; white: bystanders). (**B**) Bar graph of residues in each category. (**C**) Flowchart of hierarchy for determining the likely reason for the functional effect observed for pharmacologically important residues. (**D**) Mechanism and suggested mechanisms of action of the 82 pharmacologically important residues. The first column displays the total number of residues in each class, the second column the number of residues considering the overlap with categories of higher importance (see hierarchy of assignment of function in C), and finally the running sum of residues explained. For the remaining 28 residues, potential mechanisms are suggested on the right (inset).

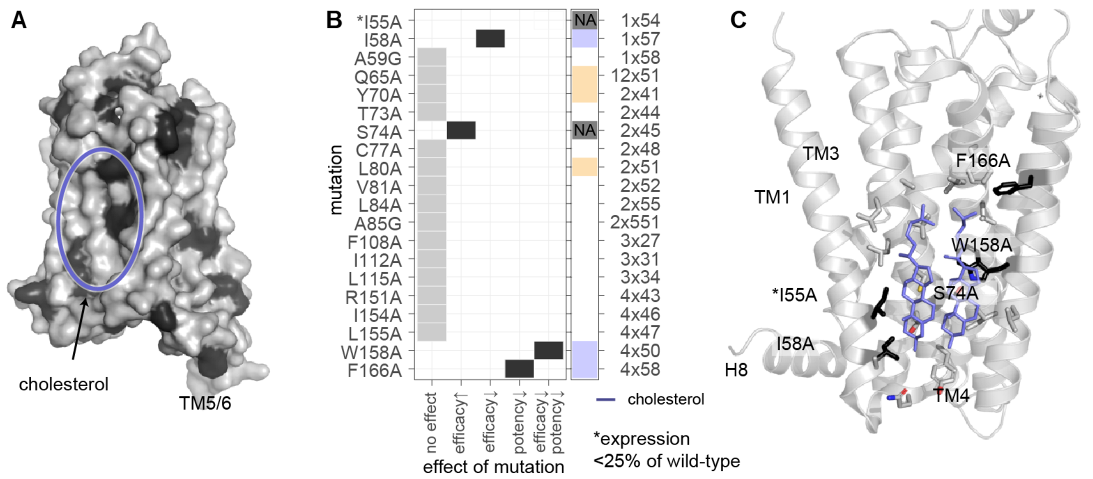

Fig. S12. Cholesterol binding site, effect of mutations, and residue classification. (A) Cholesterol bindings site marked on the structure of the β2AR (PDB ID: 5X7D), darker surfaces indicate residues that affected pharmacology upon mutation. **(B)** Effect of mutations in the cholesterol binding site on Gs signaling in response to Gs and classification of residues (right; white: bystander, light orange: passenger, light violet: modulator, grey: N/A due to low abundance). S74^{2x45} contacts cholesterol, and substitution to Alanine does not reduce but increases efficacy, possibly by influencing cholesterol binding or constitutive receptor activity. **(C)** Cholesterol-receptor interaction site with cholesterol and side chains of receptor residues shown as sticks.

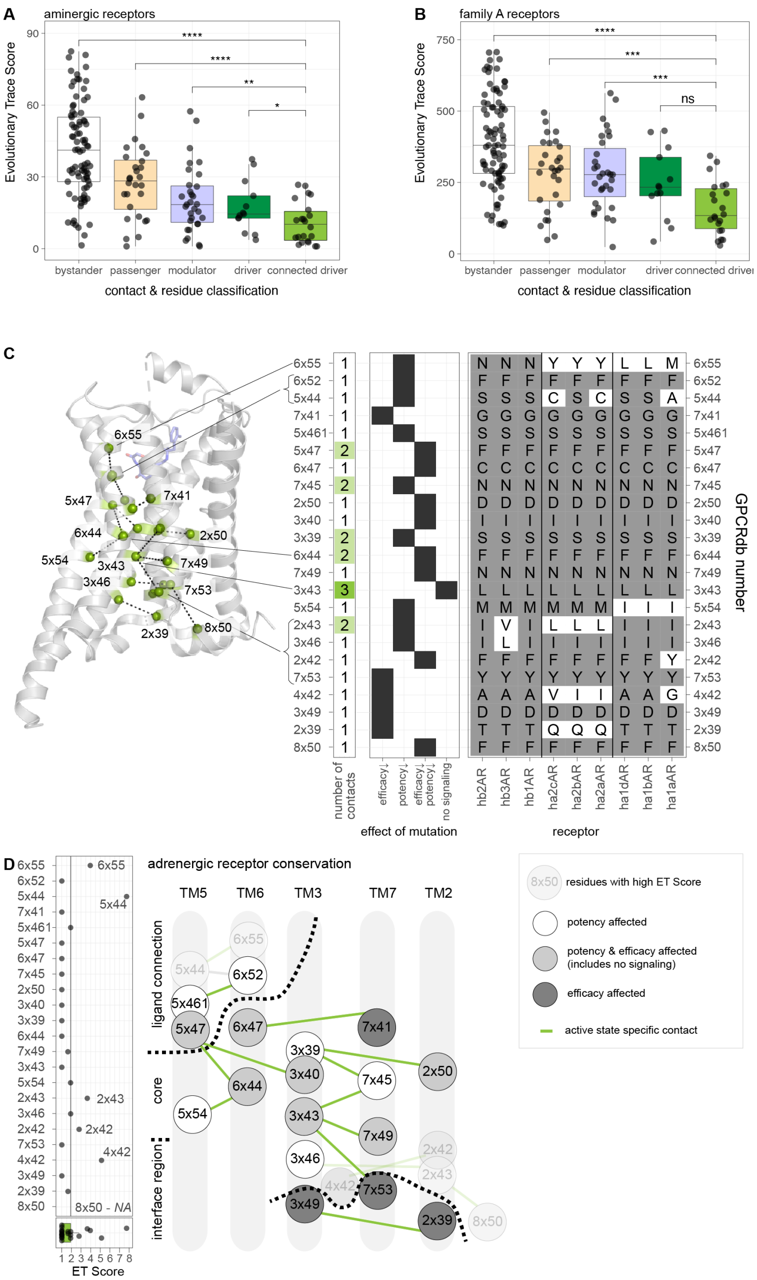

Fig. S13. Evolutionary trace score analysis of amino acid conservation of residue classes and the allosteric network. (**A**) Evolutionary Trace score analysis for 547 aminergic receptor sequences. Groups were compared using a Wilcoxon test (* p ≤ 0.05, ** p ≤ 0.01, *** p ≤ 0.001, **** p ≤ 0.0001, ns: non-significant) (**B**) Evolutionary Trace score analysis for 5105 family A GPCR sequences (not limited to human sequences). (**C**) Position of driver mutations shown on the active, G protein-bound structure (PDB ID: 3SN6), number of driver contacts made for each residue listed (green background), effect of the mutation on signaling and conservation in adrenergic receptors. (**D**) ET scores for adrenergic receptors for the connected driver residues, mapped to the allosteric network (least-conserved residues of the network shown as translucent shaded circles). Higher ET score means less conserved and lower ET score means more conserved.
