# Supplementary material for: Molecular determinants of ligand efficacy and potency in GPCR signaling
Source: Science. Author manuscript; Available in PMC 2024 Jan 16. (PMC7615523; doi:10.1126/science.adh1859)
Supplement: Supplementary Material Table Captions [file EMS193248-supplement-Supplementary_Material_Table_Captions.docx]

Table S1. Detailed data for all mutants of the β2-adrenergic receptor described in this study. For an explanation of the variables, see the ‘Explanation’ sheet.

Table S2. Active and inactive state-specific contacts. The table lists residue numbers, mutations made, GPCRdb numbers for each residue, the expression level of each mutation, the effect of mutation on the Gs biosensor, and the classification of each residue.

Table S3. List of primers used for generating mutations. For each mutation that was made, primer characteristics are listed. This includes the number of the amino acid (aa) that was mutated, the primer type (F (forward) or R (reverse)), the annealing region (mismatches shown as X, the codon to be mutated shown in upper case letters), the primer sequence length, melting temperature before and after the codon was mutated (Tm and Tmfull), the GC clamp (3 being best, options with GC clamps of 1 were excluded at the primer design stage), the number of bases of overlap between forward and reverse primers (AnnLenF), the GC content and the sequence that was ordered. Primers were designed using the program AAScan (see Methods).
